# *Staphylococcus aureus* Penicillin-Binding Protein 2 Can Use Depsi-Lipid II Derived from Vancomycin-Resistant Strains for Cell Wall Synthesis

**DOI:** 10.1002/chem.201301074

**Published:** 2013-07-19

**Authors:** Jun Nakamura, Hidenori Yamashiro, Hiroto Miya, Kenzo Nishiguchi, Hideki Maki, Hirokazu Arimoto

**Affiliations:** [a] Dr. J. Nakamura, H. Miya, Prof. Dr. H. Arimoto Graduate School of Life Sciences, Tohoku University,2-1-1 Katahira, Aoba-ku, Sendai, 980-8577 (Japan), Fax: (+81) 0-22-217-6204; [b] H. Yamashiro, Dr. K. Nishiguchi, Dr. H. Maki Medicinal Research Laboratories, Shionogi & Co., Ltd.,3-1-1 Futaba-cho, Toyonaka, Osaka, 561-0825 (Japan)

**Keywords:** antibiotics, enzymes, lipids, peptides, vancomycin

## Abstract

Vancomycin-resistant *Staphylococcus aureus* (*S. aureus*) (VRSA) uses depsipeptide-containing modified cell-wall precursors for the biosynthesis of peptidoglycan. Transglycosylase is responsible for the polymerization of the peptidoglycan, and the penicillin-binding protein 2 (PBP2) plays a major role in the polymerization among several transglycosylases of wild-type *S. aureus.* However, it is unclear whether VRSA processes the depsipeptide-containing peptidoglycan precursor by using PBP2. Here, we describe the total synthesis of depsi-lipid I, a cell-wall precursor of VRSA. By using this chemistry, we prepared a depsi-lipid II analogue as substrate for a cell-free transglycosylation system. The reconstituted system revealed that the PBP2 of *S. aureus* is able to process a depsi-lipid II intermediate as efficiently as its normal substrate. Moreover, the system was successfully used to demonstrate the difference in the mode of action of the two antibiotics moenomycin and vancomycin.

## Introduction

Drug resistance is an unavoidable consequence of the clinical use of antibiotics. Particularly, vancomycin-resistant strains among enterococci and *Staphylococcous aureus* (*S. aureus*) are of serious concern, because vancomycin is sometimes the only available antibiotic left for severe infections caused by multi-resistant gram-positive bacteria. Vancomycin resistance was initially identified in enterococci in the 1980s^1^ and is currently widely spread all over the world.^2^ For example, a report indicated that 76 % of clinically isolated *Enterococcus faecium* in intensive care units in the USA were vancomycin resistant.^3^ Although much less frequent than enterococci, horizontal transfer of the resistant gene from enterococci to *Staphylococcus aureus*,^4^ as reported in the USA,^5^ India,^6^ and Iran,^7^ may become an even more serious threat because of the more harmful nature of *S. aureus* as a nosocomial pathogen.^8^

**Figure 1:**
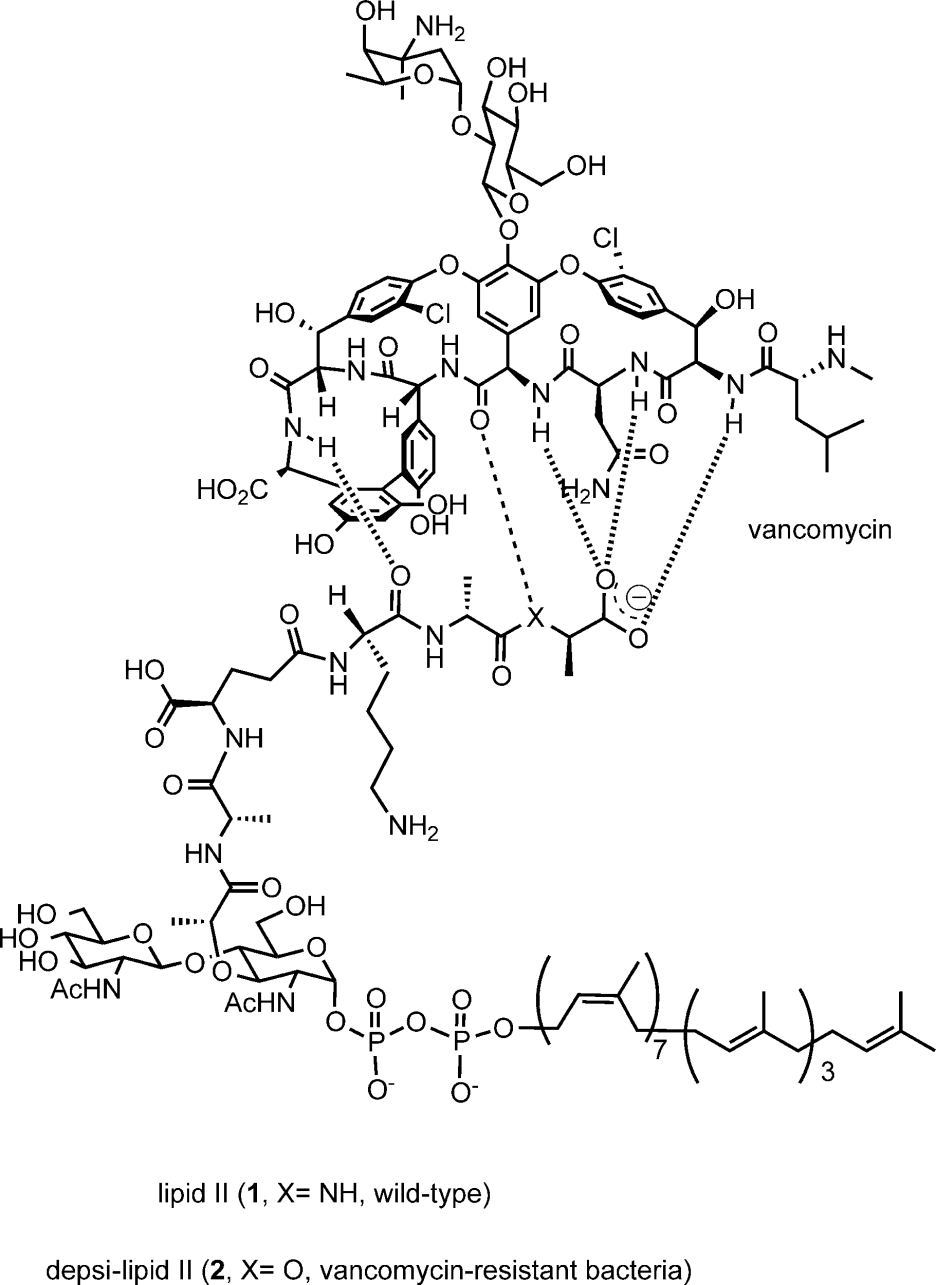
Structure of bacterial cell wall building blocks, wild-type lipid II (1) and depsi-lipid II (2), and their mode of interaction with vancomycin.

Vancomycin owes its characteristic antibacterial action to the binding with the d-alanyl-d-alanine terminal (d-Ala-d-Ala terminal, Figure 1) of bacterial cell wall intermediates.^9^ However, in the most resilient class of vancomycin-resistant bacteria (VanA and VanB phenotypes), the d-Ala-d-Ala terminal is replaced with d-alanyl-d-lactate (d-Ala-d-Lac).^10^ Vancomycin lacks the ability to bind to this ester-containing peptide (depsipeptide), and thus does not inhibit the bacterial cell wall biosynthesis. Under these circumstances, efforts have been made to develop novel antibacterial agents against vancomycin-resistant strains.^11^

In vitro reconstitution of the bacterial biosynthesis of macromolecules can serve as a valuable tool for the rational design of novel antibacterial candidates. Peptidoglycan is the major constituent of bacterial cell wall, and its biosynthesis involves a number of successive enzymatic transformations. We have recently reported a cell-free assay that reconstitutes the late stage of the biosynthesis of peptidoglycan in vancomycin-resistant *S. aureus* (VRSA).^12^

**Figure 2:**
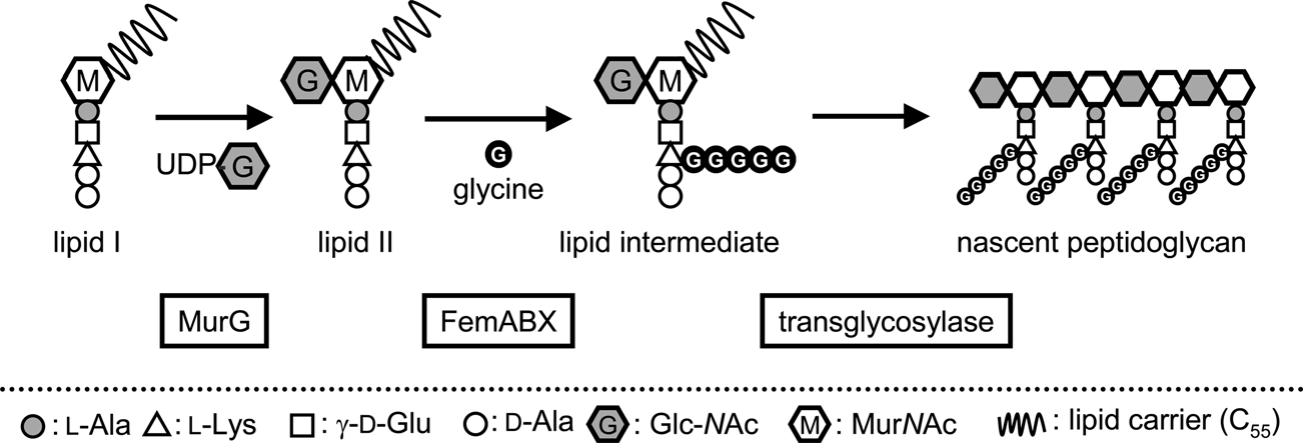
Late-stage cell wall synthesis of *S. aureus*. In vancomycin-resistant *S. aureus*. The d-Ala-d-Ala-terminal of the pentapeptide moiety is replaced with d-Ala-d-Lac. M=Mur*N*Ac; G=*N*-acetylglucosamine (Glc*N*Ac); MurG=*N*-acetylglucosamyl transferase; FemA, FemB, and FemX: peptidyltransferases.

This in vitro assay uses cell membrane of *S. aureus* that contains enzymes involved in the transformations shown in Figure 2. It provides nascent peptidoglycan from UDP-Mur*N*Ac-pentapeptide (vancomycin-susceptible model; UDP=uridine diphosphate, Mur*N*Ac=*N*-acetylmuramic acid) or UDP-Mur*N*Ac-pentadepsipeptide (resistant model). The modes of action of the vancomycin derivatives were analyzed by using this assay, and differences were successfully shown for a lipoglycopeptide^12^ and vancomycin dimers in the resistant model.^13^ However, a limitation of this assay is that inhibition by antibacterials is provided as a general effect without information on the specific action at each enzymatic step.

*S. aureus* penicillin-binding protein 2 (PBP2) is a bifunctional enzyme that catalyzes the peptidoglycan polymerization (transglycosylation, Figure 2) and its subsequent cross-linking (transpeptidation). Inhibitors of the transglycosylation are therefore considered as promising antibacterial candidates. Although PBP2 is reported to play a major role in transglycosylation of wild-type *S. aureus*, this strain has several other enzymes containing a transglycosylase domain, such as PBP1,^14^ Mgt, and SgtA.^15^ Moreover, all isolated VRSA are methicillin resistant and have the PBP2a isozyme required for methicillin resistance. Currently, there is only scant evidence for the contribution of each transglycosylase to the cell wall synthesis of vancomycin-resistant strains. Identification of the transglycosylase(s) involved in cell wall synthesis of VRSA is thus an important step for the discovery of novel antibacterial agents against VRSA.

**Figure 3:**
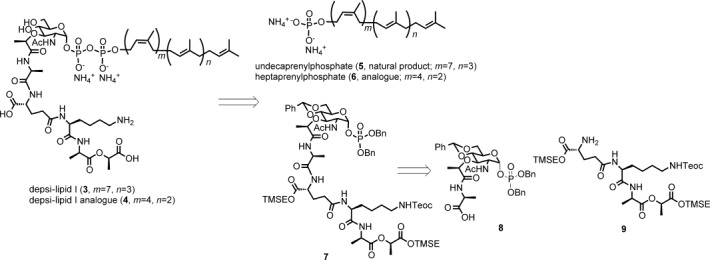
Retrosynthetic analysis for depsi-lipid I and its analogue. Bn=benzyl, Teoc=2-(trimethylsilyl)ethoxycarbonyl, TMSE=2-(trimethylsilyl)ethyl, Ac=acyl.

Here, we describe a reconstitution of PBP2 reaction by using a recombinant *S. aureus* enzyme and its substrates, lipid II (wild-type) and depsi-lipid II analogues (VRSA). Depsi-lipid I (**3**) and its analogue (**4**, Figure 3) were prepared by total synthesis. The analogue **4** was converted into a depsi-lipid II analogue by enzymatic addition of an *N*-acetylglucosamine. Kinetic analysis of the transglycosylation revealed that *S. aureus* PBP2 can process a depsi substrate with an efficiency similar to that for processing a normal substrate. The results of this study also show that cell-free peptidoglycan polymerization with a depsi substrate can shed light on the mode of action of cell wall-targeting antibiotics.

## Results and Discussion

**Total synthesis of depsi-lipid I and its analogue**: The first major obstacle in this study was the supply of depsi-lipid intermediates, 10a that is, depsi-lipid I (Figure 3) and depsi-lipid II (Figure 1), as mandatory substrates for MurG and PBP2 reactions (Figure 2). These depsi-lipid intermediates are common to VRSA and vancomycin-resistant enterococci (VRE). Although, as previously reported,^12^ isolation of cell-wall precursors in the early stage of the biosynthesis of peptidoglycan is possible, all precursors are lipidated with undecaprenyl-pyrophosphate (C55) in the late stage of the synthesis of peptidoglycan. Purification of these cell-wall precursors from natural resources has been proven to be difficult (even for wild-type lipid intermediates),^16^ and chemical synthesis is currently the only way to obtain these precursors.^17^ The total synthesis of wild-type lipid I with a d-Ala-d-Ala terminal was reported by VanNieuwenhze et al.,^18^ and that of lipid II has been reported by Schwartz et al.^19^ and VanNieuwenhze et al.^20^ Furthermore, Kahne et al. synthesized a series of lipid intermediate analogues carrying a truncated polyisoprenyl chain and demonstrated that the analogue with a heptaprenyl subunit (C35) had excellent physical properties for application in cell-free transglycosylation catalyzed by the PBP enzyme.^21^

Chemical synthesis of cell-wall precursors derived from vancomycin-resistant bacteria has rather been limited. Wong et al. synthesized an early precursor, UDP-MurNAc-depsipentapeptide (UDP-*N*-acetylmuramyl-l-Ala-d-Glu-l-Lys-d-Ala-d-Lac), which was then converted to nascent peptidoglycan by using crude bacterial enzymes.^22^ Because lipidated depsipeptide intermediates could not be easily isolated in the enzymatic transformation, we initiated our study by chemically synthesizing depsi-lipid I (**3**) and its analogue **4** with a shorter isoprenyl unit. Our retrosynthetic strategy is outlined in Figure 3. As the depsi intermediate involves a hydolytically labile carboxyl ester linkage, the synthesis^18^ of the wild-type lipid I by using base-cleavable protecting groups could not be directly applied to the synthesis of depsi-lipid I. Instead, the trimethylsilylethyl ester was used. This mildly cleavable protecting group was used by Walker et al. in the synthesis of lipid intermediate analogues,21a and then by Wong et al. in the synthesis of UDP-Mur*N*Ac-depsipentapetide.^22^

There are two approaches to the preparation of wild-type lipid intermediates. One connects the protected Mur*N*Ac with the pentapeptide,^18^, ^23^ and the other connects the protected Mur*N*Ac-l-Ala with the remaining tetrapeptide.^19^, ^20^ In this study, we adopted the latter strategy by using a tetradepsipeptide.

**Figure 4:**
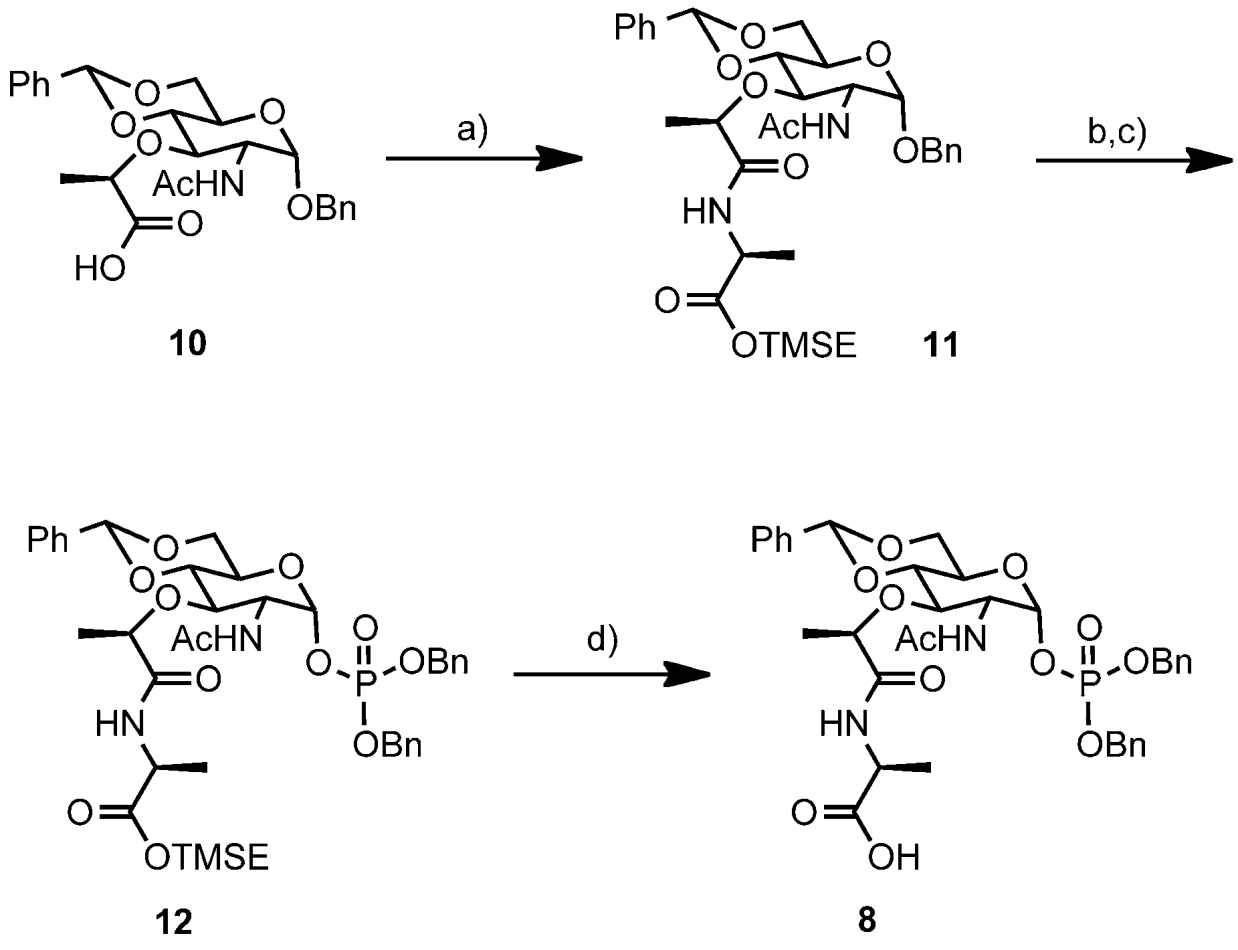
Synthesis of the phosphomuramyl peptide 8. Reagents and conditions: a) l-alanine-2-(trimethylsilyl)ethyl ester, diisopropylethylamine (DIPEA), 1-hydroxybenzotirazole (HOBt), benzotriazolyl-1-oxy-tripyrrolidinophosphonium hexafluorophosphate (PyBOP), THF/CH_2_Cl_2_, RT, 59 %; b) 10 % Pd/C, H_2_ gas, MeOH/EtOAc, RT, 83 %; c) 1 *H*-tetrazole, dibenzyl-*N*,*N*-diisopropylphosphoramidite, −40 to −10 °C, then *meta*-chloroperbenzoic acid (*m*-CPBA), −40 °C, CH_2_Cl_2_, 56 %; d) TBAF, THF, 0 °C to RT, 92 %.

**Synthesis of phosphoMur*N*Ac-depsipeptide**: Our synthesis began with the addition of l-alanine trimethylsilylethyl ester^24^ to the known Mur*N*Ac derivative **10** (Figure 4).^19^ Selective deprotection of the benzyl ether in the presence of benzylidene acetal was achieved by catalytic hydrogenation in EtOAc/MeOH (1:1). This modification significantly facilitated our synthesis. The protected glycosyl monophosphate was introduced by using the phosphoramidite method. Comparison of ^1^H and ^13^C NMR data of **12** was in good agreement with the results reported by Schwartz et al.^19^ Removal of the 2-trimethylsilylethyl ester by tetrabutylammonium fluoride (TBAF) provided the carboxylic acid **8**.

**Figure 5:**
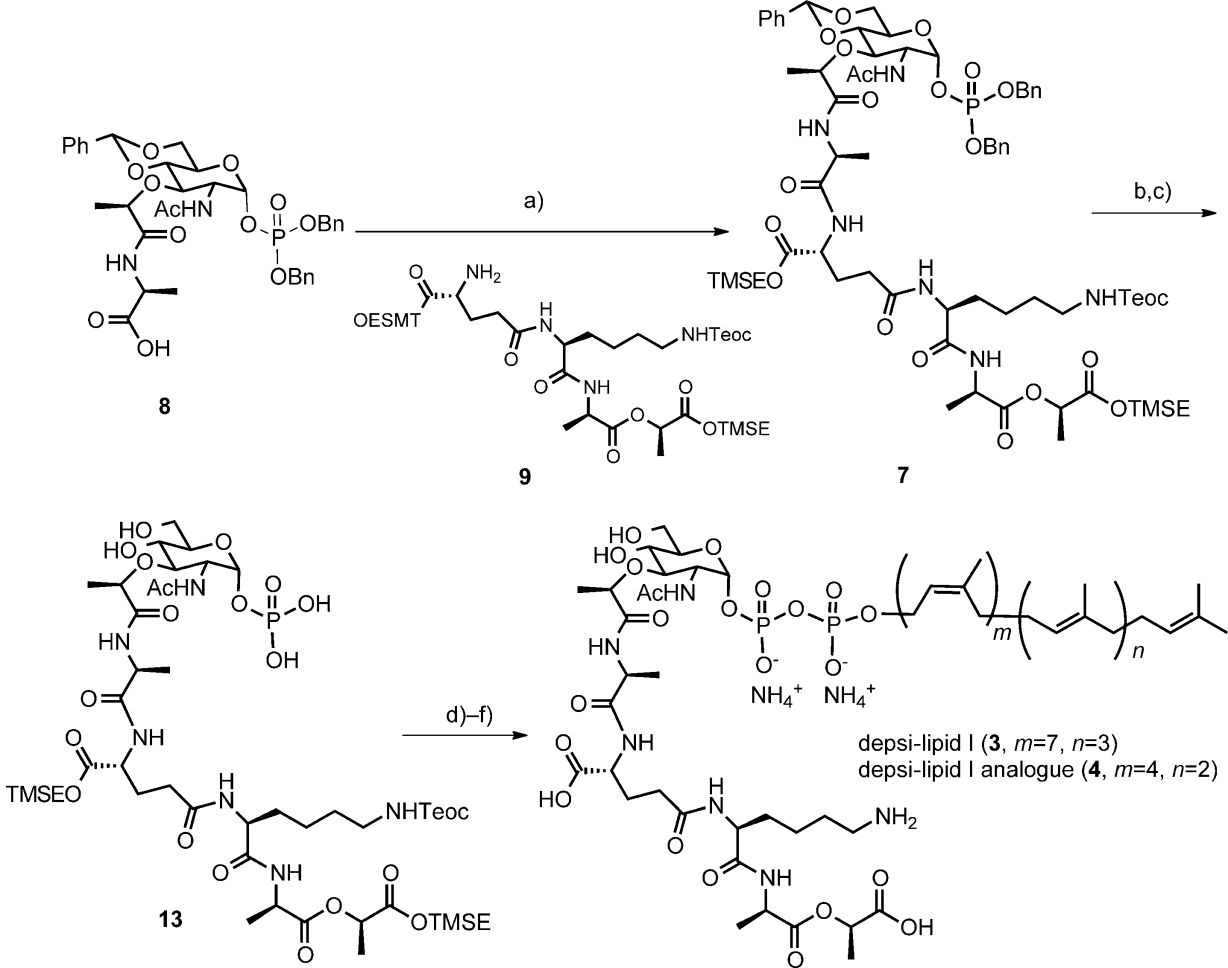
Synthesis of the depsi-lipid I and its C35 analogue. Reagents and conditions: a) compound 9, *O*-(7-azabenzotriazol-1-yl)-tetramethyluroium hexafluorophosphate (HATU), DIPEA, DMF, 0 °C, 1 h, 81 %; b) 10 % Pd/C, H_2_ gas, MeOH, RT, 30 min, 94 %; c) 80 % AcOH(aq), RT, 4 d, 91 %; d) pyridine, *N*,*N*′-carbonyldiimidazole (CDI), THF/DMF (4:1), RT, 3 h, then MeOH; undecaprenylphosphate ammonium salt 5 or 6, 1 *H*-tetrazole, THF/DMF (4:1), RT, 2 d, (78 %); f) TBAF, THF/DMF, 0 °C to RT, 2 d, 23 % (depsi-lipid I, 3) or 34 % (depsi-lipid I analogue 4).

The tetradepsipeptide **9** (Figure 5) was prepared by solution-phase synthesis by using protected amino acids and lactate (Figure S1 in the Supporting Information). The trimethylsilylethyl lactate (d-Lac-OTMSE) was synthesized from the methyl lactate by using a patent procedure.^25^ Cbz-l-Lys(Teoc)-OH (Cbz=carbobenzyloxy) was prepared from Cbz-l-Lys by using *N*-[2-(trimethylsilyl)ethoxycarbonyloxy]succinimide. Cbz-d-Glu-OTMSE was synthesized from Boc-d-Glu(OBn)-OH in four steps. These protected units were assembled into the tetradepsipeptide **9** by using standard solution conditions (Figure S2 in the Supporting Information).

The carboxylate terminal of the muramic acid derivative **8** was first activated by HATU and the resulting mixture was reacted with the depsitetrapeptide **9** to give the muramyl pentadepsipeptide **7**^22^ in 81 % yield (Figure 5). Reductive removal of the two benzyl groups at the phosphate moiety, followed by acidic hydrolysis of the benzylidene acetal provided compound **13**.

**Construction of pyrophosphate linkage**: Connection of the phosphomuramylpeptide to the polyisoprenyl phosphate requires strict anhydrous conditions, and the yields are often moderate. In the proceeding synthetic studies of wild-type lipid intermediates, either a phosphosugar^18^–^20^ or a polyisoprenyl phosphate21a, 26, 27 could be activated with CDI and the remaining part was added for coupling. We decided to activate the phosphosugar **13** with CDI in the presence of pyridine. After activation, excess CDI was quenched by methanol and the solvents were removed to dryness under reduced pressure. A solution of undecaprenyl phosphate bis-ammonium salt and 1 *H-*tetrazole in DMF was added to the activated phosphosugar and the mixture was left to react at room temperature for two days. Progress of the reaction was monitored by LC-MS. The desired protected depsi-lipid I was separated by gel filtration chromatography (LH-20, MeOH), and the crude product was treated with TBAF at 0 °C to eliminate the silyl protecting groups. The reaction mixture was evaporated to dryness, and gel filtration chromatography (LH-20, MeOH) and lyophilization provided the semi-purified depsi-lipid I, which was further purified by HPLC by using a C8 column (MeOH/(NH_4_)HCO_3_(aq)) to give the desired depsi-lipid I (**3**) (2.2 mg, 96 % purity).

By using a similar protocol, the depsi-lipid I analogue **4** with a heptaprenyl (C35) group was prepared. Because the heptaprenyl monophosphite bis-ammonium salt is not commercially available, we synthesized it from nerol and farnesol in eighteen longest linear steps (Figure S3 in the Supporting Information) according to the methods of Walker et al.^23^ and Sato et al.^28^

**Preparation of the depsi- lipid II analogue as substrate for the transglycosylase reaction**: MurG is a membrane-associated enzyme that converts wild-type lipid I to lipid II (Figure 2). In this study, *Escherichia coli* (*E. coli*) MurG was purified according to the method described in a previous report by Walker et al.^23^ Briefly, MurG was overproduced in *E. coli* and purified by Ni^2+^-affinity column chromatography. After further purification by size exclusion chromatography, sodium dodecyl sulfate polyacrylamide gel electrophoresis (SDS-PAGE) analysis showed a single protein band at about 40 kDa. Walker et al. reported a reconstitution of the cell wall synthesis by using C35-lipid I and *E. coli* MurG, because wild-type lipid I with a C55-isoprenyl chain aggregates in a manner that precludes the reaction in solution.21a Thus, we used the C35-depsi-lipid I **4** in this study. The MurG reaction was carried out in a buffer containing the C35 analogue and UDP-(^14^C)-Glc*N*Ac as substrates. Progress of the reaction was monitored by incorporation of radiolabeled Glc*N*Ac into the depsi-lipid II analogue. Purification with high-performance liquid chromatography provided the radiolabeled depsi-lipid II analogue (≈2 mg, Figure S5 in the Supporting Information). The C35-lipid II with a d-Ala-d-Ala terminal (Figure S5 in the Supporting Information) was prepared as reference substrate for the enzyme assay by chemical synthesis according to a reported procedure.21a

**Transglycosylation of the radiolabeled depsi-lipid II by**­ ***S. aureus***­ **PBP2**: By using the radiolabeled product of the MurG reaction, we next investigated the polymerization of peptidoglycan with purified *S. aureus* PBP2 enzyme. A full-length PBP2 of *S. aureus* RN4220 strain^29^, ^30^ was prepared as previously described. The C35-lipid II containing a d-Ala-d-Ala terminal was used for the vancomycin-susceptible model, and the C35-depsi-lipid II with a d-Ala-d-Lac terminal was used for the resistant model. The activity of PBP2 with each substrate was evaluated by measurement of the peptidoglycan incorporated radioactivity by using thin-layer chromatography (TLC). The obtained kinetic parameters *K*_m_, *V*_max_, and *k*_cat_ of the PBP2 reaction are shown in Table 1.

**Table 1:** Kinetic parameters of the PBP2^[a]^ reaction.^[b]^

	Susceptible model	Resistant model
*V*_max_ [μm s^−1^]	(0.91±0.16)	(1.2±0.56)
*K*_m_ [μm]	(11.0±1.6)	(6.0±2.2)
*k*_cat_ [10^−3^ s^−1^]	(2.5±0.4)	(3.2±1.5)

[a] *S. aureus* PBP2. [b] C35-lipid II was used.

The kinetic parameters for the *S. aureus* PBP2 reaction were previously determined by Walker et al. at pH 5.0 by using the same C35-lipid II, which corresponds to that of our susceptible model.^30^ In our susceptible model, the reaction was slightly slower at pH 7.0, but the obtained parameters were within 6-fold those obtained by Walker et al. at pH 5.0. This may be rationalized by considering the reported optimum pH value of *S. aureus* PBP2 for wild-type lipid II as substrate (pH 4.5–5.5).

With the C35-depsi-lipid II substrate, transglycosylation at pH 7.0 was faster and had a *K*_m_ value comparable to that of C35-lipid II, which indicates that depsi-lipid II can also be an excellent substrate of PBP2.

**Figure 6:**
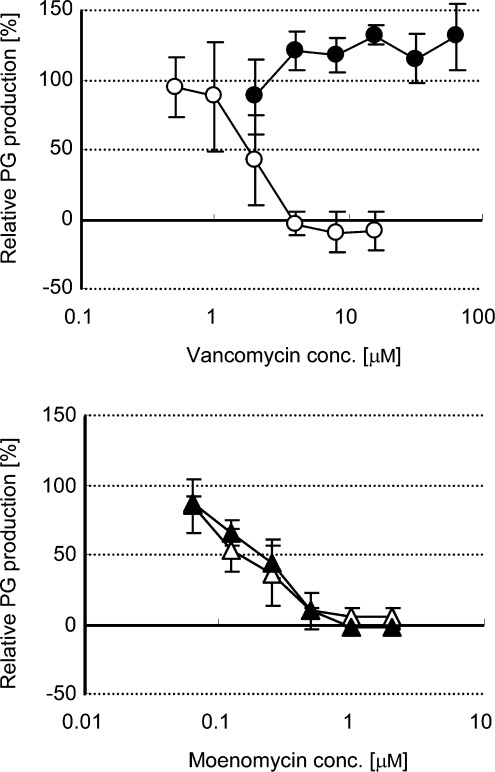
Inhibitory effect of vancomycin and moenomycin on the PBP2 reaction. ○ and ▵=lipid II, • and ▴=depsi-lipid II. PG: peptidoglycan.

**Characterization of modes of action of the antibiotics within the reconstituted PBP2 reaction**: We next examined whether the in vitro PBP2 reaction can be used to show differences in the action of the antibiotics. Among all enzymatic transformations in the late-stage cell wall biosynthesis, PBP2 is unique in that it works extracellularly. Some antibiotics, such as vancomycin do not penetrate the bacterial membrane and are effective only at the outer surface of the bacterial membrane. This indicates that PBP is a good target for antibacterial drugs. In our susceptible and resistant models of the PBP2 reactions, two cell-wall-targeting antibiotics, that is, vancomycin and moenomycin, exhibited distinct inhibitory patterns (Figure 6 A and Table 2). Moenomycin suppressed the peptidoglycan polymerization regardless of the use of normal or depsi-lipid II analogues. Vancomycin, on the other hand, suppressed the peptidoglycan polymerization only when normal lipid II analogue was used. This is because, as described earlier, vancomycin is a substrate-binding antibiotics that cannot bind to the d-Ala-d-Lac terminal of depsi-lipid II. The d-Ala-d-Ala terminal of the normal lipid intermediate is a crucial motif for the activity of vancomycin. In contrast, moenomycin inhibits the PBP2 reaction by directly binding to the enzyme,^31^ and thus is able to suppress in vitro transglycosylation regardless of the substrate used.^32^

**Table 2:** Inhibitory effect of vancomycin and moenomycin on the PBP2^[a]^ reaction.

	IC_50_ [µm]
	susceptible model^[b]^	resistant model^[c]^
vancomycin	(1.52±0.52)	>64
moenomycin	(0.16±0.08)	(0.19±0.03)

[a] *S. aureus* PBP2. [b] C35-lipid II was used. [c] C35-depsi-lipid II was used.

## Conclusion

We have shown in this study that PBP2 is able to catalyze the transglycosylation of a VRSA peptidoglycan precursor. Chemical synthesis of depsi-lipid I and its analogue established the basis of this study.

The clinically important classes of vancomycin-resistant bacteria (VanA and VanB phenotypes) induce expression of resistance-related enzymes that build the UDP-Mur*N*Ac-pentadepsipeptide with the d-alanyl-d-lactate terminus as a modified peptidoglycan precursor. Enzymes responsible for the conversion of this precursor into the peptidoglycan are unaltered and seem to accept modified substrates. However, VRSA has three PBPs (PBP1, PBP2, and PBP2a) with a transglycosylase domain and monofunctional transglycosylases (e.g., Mgt and SgtA). There has only been scant evidence for the contribution of each transglycosylase to the polymerization of the abnormal depsipeptide-containing precursor. The current study indicates that depsi-lipid II can also be an excellent substrate of PBP2. Nonetheless, we do not eliminate the possibility that other transglycosylases could also use depsi-lipid II. Actually, Pinho et al. reported that monofunctional transglycosylases are not essential for the cell-wall synthesis of vancomycin-susceptible *S. aureus*, whereas in the absence of PBP2 activity, the monofunctional transglycosylase Mgt can be the sole enzyme for the peptidoglycan polymerization.^15^ These observations indicate the need for additional studies with depsi-lipid II to examine whether Mgt and other transglycosylases are able to process the VRSA cell-wall precursor. If monofunctional transglycosylases were able to use the precursor, dual inhibitors of PBP2 and the monofunctional transglycosylases might be required for an efficient suppression of the cell wall synthesis of VRSA.

We also demonstrated in this study that the reconstituted PBP2 reaction with a depsi substrate is an indispensible tool in the search for antibacterials against VRSA. Antibiotics that require d-Ala-d-Ala binding (i.e., vancomycin) did not suppress the PBP2 reaction with depsi-lipid II, whereas antibiotics with direct interaction with the PBP2 enzyme did. Reconstitution of the PBP2 reaction with a d-Ala-d-Ala-containing normal substrate has actually been reported.^33^ However, the use of a depsipeptide-containing substrate allows detailed evaluation of the transglycosylase inhibitor–PBP2 interaction without interference of the binding between the inhibitor and the PBP2 substrate containing d-Ala-d-Ala.^34^

In conclusion, we performed in this study the first chemical synthesis of the modified cell-wall precursors depsi-lipid I (**3**) and its analogue **4**. These precursors enabled the reconstitution of the very last stage of the biosynthesis of peptidoglycan. The in vitro PBP2 reaction by using both normal and modified peptidoglycan precursors would open a new way for understanding the mode of action of anti-VRSA/VRE compounds.

## Experimental Section

**General methods**: All reagents and solvents were used as purchased from commercial suppliers. Undecaprenyl phosphate ammonium salt was purchased from Larodan Fine Chemicals AB (Malmö, Sweden). [^14^C]-UDP-Glc*N*Ac was obtained from American Radiolabeled Chemicals, Inc. (Saint Louis, USA). All reactions were carried out at room temperature unless otherwise noted. Mass spectra were obtained by using a JEOL LMS-700 mass spectrometer (FAB MS) and a Bruker Daltonics microTOF-TSfocus (LC-ESI-TOF MS). LC was performed on an Agilent 1100 Series, by using an Imtakt UNISON UK 3C_8_ column (150×2 mm) under the following conditions: flow rate=0.2 mL min^−1^, 65 to 95 % acetonitrile/water containing 0.1 % formic acid, duration=5 min, temperature=40 °C, and detection at *λ*=214, 254, 280, and 190–400 nm. The monoisotopic mass was used for calculation of the exact mass (C=12.0000, H=1.0078, O=15.9949, N=14.0031, Cl=35.9689). Nuclear magnetic resonance spectra were recorded on a JEOL ECA600 at 600 MHz, a Varian Unity INOVA 500 at 500 MHz, and an INOVA 600 at 600 MHz. Chemical shifts (*δ*) are reported in parts per million [ppm] in the ^1^H NMR spectra relative to the residual solvent peak of tetramethylsilane (0 ppm), CHCl_3_ (7.26 ppm), CHD_2_OD (3.31 ppm), (CHD_2_)(CD_3_)SO (2.5 ppm), and water (3.33 ppm in [D_6_]DMSO), and in the ^13^C NMR spectra relative to the residual solvent peak of CHCl_3_ (77.16 ppm). ^31^P NMR spectra were recorded using 85 % H_3_PO_4_ (0 ppm) as an external standard. Coupling constants are given in Hertz [Hz]. Thin-layer chromatography (TLC) was performed by using Merck silica gel 60 F254 precoated plates (0.25 mm). Flash chromatography was carried out by using 60–230 mesh silica gel (Kanto Chemical, silica gel 60N). The purity of the compounds was assessed by HPLC under the following conditions: method I: Cosmosil 5C_18_-AR-II (150×4.6 mm, Nacalai Tesque), 15 to 100 % acetonitrile/water containing 0.1 % trifluoroacetic acid (TFA), flow rate=1.0 mL min^−1^, duration=10 min, temperature=30 °C, detection at *λ*=280 nm; method II: Unison UK-C_8_ (150×4.6 mm, particle size 3 μm, Imtakt), 80 to 95 % acetonitrile/water containing 0.1 % NH_4_HCO_3_, flow rate=1.0 mL min^−1^, duration=10 min, room temperature, UV detection at *λ*=214 nm).

**Synthesis compound 11**: l-Ala-OTMSE (0.312 g, 0.1.65 mmol), DIPEA (0.6 mL, 3.4 mmol), PyBOP (0.98 g, 1.9 mmol), and HOBt (0.23 g, 1.7 mmol) were added to a solution of benzyl-4,6-*O*-bezylidene-Mur*N*Ac (**10**) (0.81 g, 1.7 mmol) in dry THF/dry dichloromethane (1:1) (20 mL). The mixture was stirred at room temperature for 5 h and partitioned between a water layer (50 mL) and an organic layer (ethyl acetate, 5×50 mL). The combined organic layers were washed with brine (100 mL), dried over anhydrous magnesium sulfate, and evaporated to give the crude compound **11** as an oil. Purification by silica gel column chromatography (chloroform/methanol 40:1) yielded **11** as a colorless solid (0.64 g, 59 %). *R*_f_=0.68 (chloroform/methanol=9:1); ^1^H NMR (500 MHz, CDCl_3_, 20 °C): *δ*=7.47–7.32 (complex multiplet (comp.), 10 H), 6.93 (d, *J*=7 Hz, 1 H), 6.28 (d, *J*=8.5 Hz, 1 H), 5.56 (s, 1 H), 4.98 (s, 1 H), 4.72 (d, *J*=12 Hz, 1 H), 4.50 (d, *J*=12 Hz, 1 H), 4.46 (m, 1 H), 4.28 (dt, *J*=9.5, 5.5 Hz, 1 H), 4.27–4.19 (comp., 3 H), 4.13 (q, *J*=7 Hz, 1 H), 3.88 (dt, *J*=9, 4.5 Hz, 1 H), 3.79–3.67 (comp., 3 H), 1.94 (s, 3 H), 1.42 (d, *J*=7.5 Hz, 3 H), 1.39 (d, *J*=7 Hz, 3 H), 1.01(m, 2 H), 0.03 ppm (s, 9 H); ^13^C NMR (125 MHz, CDCl_3_, 20 °C): *δ*=172.8, 172.6, 170.4, 137, 136.8, 128.9, 128.6, 128.3, 128.1, 125.1, 101.4, 97.5, 81.7, 78.2, 77.5, 70.1, 68.8, 63.7, 63.1, 53, 48, 23.4, 19.4, 17.9, 17.2, −1.55 ppm; HRMS (FAB) calcd for C_33_H_47_N_2_O_9_Si^+^: 643.3051 [*M*+H]^+^; found: 643.3055 [*M*+H]^+^.

**Synthesis of compound 12**: Methanol (13 mL) and 10 % Pd/C (1.5 g, 150 % w/w) were added to benzyl-4,6-*O*-benzylidene-Mur*N*Ac-l-Ala-OTMSE (**11**) (1.0 g, 1.6 mmol) dissolved in ethyl acetate (13 mL). Selective removal of the benzyl group was succeeded by catalytic reduction under a hydrogen atmosphere. After stirring at room temperature for 12 h, the reaction mixture was filtered to remove the Pd/C catalyst and then evaporated to give the crude hemiacetal product. Purification of the crude material by silica gel column chromatography (chloroform/methanol 20:1 to 5:1) provided the hemiacetal as a colorless amorphous (0.73 g, 83 %). *R*_f_=0.37 (chloroform/methanol 9:1); ^1^H NMR (500 MHz, CDCl_3_, 20 °C): *δ*=7.46–7.36 (comp., 5 H), 7.01 (d, *J*=7 Hz, 1 H), 6.87 (d, *J*=6.5 Hz, 1 H), 5.56 (s, 1 H), 5.37 (s, 1 H), 4.51 (quin., *J*=7 Hz, 1 H), 4.27–4.13 (comp., 5 H), 4.04 (dt, *J*=9.5, 4.5 Hz, 1 H), 3.75 (m, 2 H), 3.63 (t, *J*=9.5 Hz, 1 H), 2.72 (s, 1 H), 2.02 (s, 3 H), 1.84 (s, 1 H), 1.43 (d, *J*=7 Hz, 3 H), 1.41 (d, *J*=7 Hz, 3 H), 1.03 (m, 2 H), 0.039 ppm (s, 9 H); ^13^C NMR (125 MHz, CDCl_3_, 20 °C): *δ*=173.3, 172.7, 171.3, 137.1, 128.9, 128.1, 125.9, 101.2, 91.1, 81.7, 77.8, 76.9, 68.8, 63.8, 62.5, 53.7, 48, 23.2, 19.3, 17.7, 17.1, −1.61 ppm; HRMS (FAB) calcd for C_26_H_41_N_2_O_9_Si^+^: 553.2581 [*M*+H]^+^; found: 553.2579 [*M*+H]^+^.

The hemiacetal product of the above-described reaction (1.3 g, 2.4 mmol) and 1 *H*-tetrazole (1 g, 14 mmol) were azeotroped with toluene/dichloromethane (1:1) (2×30 mL), and then placed under vacuum. Dry dichloromethane (120 mL) was then added and the mixture was cooled to −68 °C. After dropwise addition of dibenzyl-*N*,*N*-diisopropylphosphoramidite (2.4 mL, 7.2 mmol) over 5 min, the reaction mixture was warmed to −40 °C over 2 h, at which point the starting material was no longer evident. The reaction mixture was then cooled to −65 °C and *m*-CPBA (3.6 g, 22 mmol) was added. The mixture was allowed to warm to −40 °C over 1 h, and poured into ethyl acetate (0.4 L). The organic layer was successively washed with 50 % sodium sulfite(aq) (0.4 L) and brine (0.3 L), dried over anhydrous magnesium sulfate, and concentrated under vacuum to yield the crude **12** (7.8 g). Purification of the crude **12** by silica gel column chromatography (chloroform/methanol 20:1), followed by alumina column chromatography (Al_2_O_3_ activated, Nacalai Tesque, chloroform/methanol 20:1) gave compound **12** as a colorless amorphous (1.1 g, 56 %). *R*_f_=0.57 (chloroform/methanol 9:1); ^1^H NMR (500 MHz, CDCl_3_, 20 °C): *δ*=7.47–7.34 (comp., 15 H), 6.79 (d, *J*=7.5 Hz, 1 H), 6.69 (d, *J*=8 Hz, 1 H), 5.79 (dd, *J*=5, 4.3 Hz, 1 H), 5.53 (s, 1 H), 5.12 (m, 4 H), 4.5 (quin., *J*=7.5 Hz, 1 H), 4.28–4.19 (comp., 3 H), 4.15 (q, *J*=6.5 Hz, 1 H), 4.09 (dd, *J*=11, 7.8 Hz, 1 H), 3.91 (dt, *J*=10, 5 Hz, 1 H), 3.67 (q, *J*=9.6 Hz, 2 H), 3.61 (t, *J*=10 Hz, 1 H), 1.82 (s, 3 H), 1.42 (d, *J*=7.5 Hz, 3 H), 1.39 (d, *J*=6.5 Hz, 3 H), 1.01 (m, 2 H), 0.036 ppm (s, 9 H); ^13^C NMR (125 MHz, CDCl_3_, 20 °C): *δ*=172.7, 172.6, 170.8, 136.9, 135.47, 135.42, 135.25, 129.1, 128.8, 128.68, 128.66, 128.3, 127.99, 127.88, 125.9, 101.4, 96.8, 81, 77.8, 75.9, 69.8, 68.3, 64.6, 63.8, 53.2, 48, 22.9, 19.3, 18, 17.2, −1.59 ppm; HRMS (FAB) calcd for C_40_H_53_N_2_NaO_12_PSi^+^: 835.3003 [*M*+Na]^+^; found: 835.3007 [*M*+Na]^+^.

S**ynthesis of compound 8**: A solution of tetrabutylammonium fluoride (1.0 m) in THF (0.65 mL) was added to a solution of benzylphospho-4,6-*O*-benzylidene-Mur*N*Ac-l-Ala-OTMSE (**12**) (0.45 g, 0.56 mmol) in dry THF (28 mL) at 0 °C. After stirring at 0 °C for 1 h, the reaction mixture was warmed to room temperature and stirred for additional 2 h. The mixture was then evaporated under reduced pressure and the resulting residue was subjected to silica gel column chromatography (chloroform/methanol 4:1) to yield **8** as a colorless oil (0.39 mg, 99 %). *R*_f_=0.47 (chloroform/methanol 9:1); ^1^H NMR (500 MHz, CD_3_OD, 20 °C): *δ*=7.45–7.28 (comp., 15 H), 5.83 (dd, *J*=6, 4.8 Hz, 1 H), 5.57 (s, 1 H), 5.07–5.03 (comp., 4 H), 4.31–4.26 (comp., 2 H), 4.05 (m, 1 H), 3.98 (dd, *J*=8, 4 Hz, 1 H), 3.78–3.66 (comp., 4 H), 1.8 (s, 3 H), 1.34 (d, *J*=7 Hz, 3 H), 1.28 ppm (d, *J*=7 Hz, 3 H); ^13^C NMR (150 MHz, CD_3_OD, 20 °C): *δ*=176.3, 175.5, 173.9, 138.8, 136.98, 136.96, 136.9, 130, 129.89, 129.83, 129.77, 129.75, 129.2, 129.1, 127.3, 102.8, 97.8, 82.5, 78.3, 76.2, 71.1, 69.1, 66, 55.1, 55, 22.7, 19.7, 17.7 ppm; HRMS (FAB) calcd for C_35_H_42_N_2_O_12_P^+^: 713.2475 [*M*+H]^+^; found: 713.2479 [*M*+H]^+^.

**Synthesis of compound 7**: The protected tetradepsipeptide-OTMSE **9** (0.15 g, 0.20 mmol), DIPEA (1.1 mL, 6.3 mmol), and HATU (0.24 g, 0.63 mmol) were added to a solution of compound **8** (0.15 g, 0.21 mmol) in dry DMF (6 mL). The reaction mixture was stirred at room temperature for 1 h and then evaporated to give the crude product **7**, which was purified by silica gel column chromatography (chloroform/methanol 40:1) to yield **7** as a white solid (0.24 g, 81 %). ^1^H NMR (500 MHz, CD_3_OD, 20 °C): *δ*=8.47 (d, *J*=7 Hz, 1 H), 8.33 (d, *J*=6.5 Hz, 1 H), 8.26 (d, *J*=8.5 Hz, 1 H), 8.01 (m, 2 H), 7.49–7.36 (comp., 15 H), 5.86 (dd, *J*=6, 4 Hz, 1 H), 5.63 (s, 1 H), 5.13–5.09 (comp., 4 H), 5.06 (q, *J*=7 Hz, 1 H), 4.45 (t, *J*=7.5 Hz, 1 H), 4.38–4.28 (comp., 5 H), 4.2–4.15 (comp., 6 H), 4.12 (t, *J*=8.5 Hz, 2 H), 4.05 (dd, *J*=11, 3.8 Hz, 1 H), 3.84–3.73 (comp., 5 H), 3.08 (t, *J*=6.5 Hz, 2 H), 2.26 (t, *J*=6.5 Hz, 2 H), 2.21 (m, 1 H), 1.86 (s, 3 H), 1.77 (m, 1 H), 1.65 (m, 1 H), 1.47–1.43 (comp., 9 H), 1.38 (d, *J*=7 Hz, 3 H), 1.35 (d, *J*=6 Hz, 3 H), 1.03–0.95 (comp., 6 H), 0.03 (s, 18 H), 0.02 ppm (s, 9 H); ^13^C NMR (150 MHz, CD_3_OD, 20 °C): *δ*=175.4, 174.5, 174.38, 174.34, 173.6, 173.4, 172.8, 171.9, 159.1, 138.8, 136.93, 136.88, 136.84, 129.8, 129.2, 129.1, 127.3, 102.6, 97.8, 82, 78.4, 76.5, 71, 70.5, 69, 65.9, 64.7, 63.6, 58.3, 54.9, 54.7, 52.9, 50.3, 41.3, 32.6, 32.5, 30.5, 28.6, 23.9, 22.9, 19.8, 18.6, 18.4, 18.14, 18.12, 18.1, 17.3, 17.2, −1.36. −1.43, −1.45 ppm; HRMS (FAB) calcd for C_68_H_106_N_6_O_21_PSi_3_^+^: 1457.6456 [*M*+H]^+^; found: 1457.6453 [*M*+H]^+^.

**Synthesis of compound 13**: Benzylphospho-4,6-*O*-benzylidene-Mur*N*Ac-silyl-protected-pentadepsipeptide-OTMSE **7** (0.20 g, 0.14 mmol) was dissolved in methanol (3 mL) and its benzyl groups were removed by catalytic hydrogenation in the presence of 10 % Pd/C at room temperature for 30 min. The Pd/C catalyst was removed by filtration through celite and the solution was evaporated to dryness to yield the corresponding phosphoric acid (0.16 g, 94 %) as a colorless solid, which was used for the next reaction without further purification. Acetic acid in water (80 %, 10 mL) was added the obtained phosphoric acid (0.12 g, 94 μmol) and the reaction mixture was stirred at room temperature for 1.5 d at which point the reaction was complete. The mixture was diluted with toluene (100 mL) and evaporated to dryness to yield compound **13** as a colorless solid (111 mg, 91 %). ^1^H NMR (300 MHz, CD_3_OD, 20 °C): *δ*=5.28 (m, 1 H), 4.68 (q, *J*=4.4 Hz, 1 H), 4.58–4.50 (comp., 4 H), 4.47–4.42 (comp., 4 H), 4.35 (t, *J*=4.7 Hz, 2 H), 4.11 (br s, 2 H), 3.75 (br s, 1 H), 3.31 (s, 2 H), 2.54–2.45 (comp., 3 H), 2.29–2.17 (comp., 5 H), 2.01 (m, 1 H), 1.91 (m, 1 H), 1.71–1.65 (comp., 18 H), 1.28–1.18 (comp., 6 H), 0.10 ppm (m, 27 H); ^13^C NMR (150 MHz, CD_3_OD, 20 °C): *δ*=175.9, 174.8, 174.5, 173.5, 172.8, 172, 159.2, 109, 80.7, 78.4, 74.9, 70.6, 70.5, 64.73, 64.69, 63.6, 62.5, 54.9, 54.7, 52.9, 50.6, 41.3, 32.6, 32.4, 30.5, 28.2, 23.9, 23.1, 19.7, 18.6, 18.3, 18.2, 18.1, 17.1, 17.1, −1.39, −1.48 ppm; HRMS (LC-ESI-TOF) calcd for C_47_H_88_N_6_O_21_PSi_3_^−^: 1187.5053 [*M*−H]^−^; found: 1187.5019 [*M*−H]^−^.

**Synthesis of the depsi-lipid I analogue 4**: Dry pyridine (3.5 μL) was added to the phospho-Mur*N*Ac-silyl-protected-pentadepsipeptide-TMSE **13** (25 mg, 21 μmol) and excess pyridine was removed under reduced pressure to give the pyridinium salt. This salt was azeotropically dried with toluene/benzene (1:1, 2×1 mL) under vacuum, and the residue was dissolved in dry THF/DMF (4:1, 0.25 mL) and then added to a flask containing a solution of CDI (10 mg, 63 μmol) in dry THF/DMF (1:1, 1 mL) at 0 °C (ice/water bath) through a cannula. The whole was finally rinsed with dry THF/dry DMF (1:1, 3×0.4 mL). The reaction mixture was first allowed to warm to room temperature and then stirred for further 3 h at which point the reaction was complete. Dry methanol (3 μL) was added to the reaction mixture and the whole was evaporated to remove THF. The residue was azeotropically dried with toluene/benzene (1:1, 1 mL) to yield a solution of the activated phosphoimidazolide of **13** in DMF.

The heptaprenyl phosphate diammonium salt **6** (13 mg, 19 μmol) was azeotropically dried with toluene/benzene (1:1, 2×1 mL), and the flask was purged with argon gas. The solution of the activated phosphoimidazolide was transferred to the flask containing **6** through a cannula, and rinsed with dry THF/DMF (1:1, 3×0.3 mL). The mixture was supplemented with 1 *H*-tetrazole (6.0 mg, 83 μmol) and stirred at room temperature for 2.5 d. After removal of THF under reduced pressure, the residue was purified by gel permeation chromatography (Sephadex LH-20, GE Healthcare, 260×15 mm, methanol) to yield the silyl-protected depsi-lipid I analogue (38 mg, <21 μmol).

This analogue (25 mg) was azeotropically dried with toluene and dissolved in dry DMF (0.3 mL). TBAF (1.0 m) in THF (0.43 mL) was then added to the protected analogue at 0 °C and the reaction mixture was stirred and allowed to warm to room temperature for 2 d, at which point the reaction was complete as confirmed by LC-MS. The reaction mixture was next evaporated to dryness under vacuum and the residue was purified by gel permeation chromatography (Sephadex LH-20, GE Healthcare, 260×15 mm, methanol). The fractions containing the desired compound were combined, diluted with water, and then lyophilized overnight to yield the semi-purified depsi-lipid I analogue as a pale yellow solid (19 mg, <14 μmol). This analogue was further purified by reverse-phase HPLC (Imtakt, Unison UK-C_8_, particle size 3 μm, 250×10 mm, 80 to 95 % methanol in 0.1 % NH_4_HCO_3_(aq), duration=20 min, flow rate=3 mL min^−1^, detection at *λ*=214 nm). The eluted fractions were combined and then lyophilized overnight to give the depsi-lipid I analogue **4** as a white solid (6.8 mg, 4.7 μmol, 34 % yield, 96 % HPLC purity). The purity of this compound was assessed by analytical method II. *R_t_*=5.8 min; [*α*]
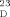
=+33° (*c*=0.10 in MeOH); ^1^H NMR (600 MHz, D_2_O, 20 °C): *δ*=6.89 (dd, *J*=7.2, 3 Hz, 1 H), 6.86 (t, *J*=6 Hz, 1 H), 6.53–6.48 (comp., 6 H), 5.92 (t, *J*=6.0 Hz, 2 H), 5.79 (q, *J*=7.4 Hz, 1 H), 5.64–5.59 (comp., 4 H), 5.33 (m, 1 H), 5.27 (d, *J*=12 Hz, 1 H), 5.19 (dd, *J*=12, 5.4 Hz, 1 H), 5.1 <0 (t, *J*=12.0 Hz, 1 H), 4.95 (t, *J*=9.0 Hz, 1 H), 4.31 (t, *J*=6.6 Hz, 2 H), 3.75–3.74 (comp., 1 H), 3.72 (t, *J*=6.3 Hz, 2 H) 3.52–3.44 (comp., 20 H), 3.42 (s, 3 H), 3.39 (m, 4 H), 3.21 (m, 3 H), 3.13 (s, 3 H), 3.07–3.06 (comp., 14 H), 3.01 (s, 3 H), 2.99 (s, 6 H), 2.86–2.84 (comp., 9 H), 2.81 ppm (d, *J*=6.6 Hz, 3 H); ^13^C NMR (150 MHz, D_2_O, 20 °C): *δ*=177.9, 176.9, 175.5, 175.4, 174.9, 173.8, 173.71, 173.69, 140.6, 136.4, 136.3, 136.2, 136, 132.1, 126.1, 126.2, 125.9, 12.5, 125.4, 123.6, 123.5, 100.1, 96.3, 81.8, 79.1, 75.3, 73.1, 70.2, 63.6, 62.6, 55.7, 54.9, 54.4, 50.4, 49.9, 40.88, 40.85, 40.5, 33.32, 33.26, 32.9, 32.8, 32.5, 30.4, 28.4, 27.8, 27.7, 27.6, 27.5, 25.96, 25.93, 23.85, 23.83, 23.79, 23.77, 23.75, 23.72, 23.68, 19.3, 18.8, 17.9, 17.8, 16.9, 16.16, 16.14 ppm; ^31^P NMR (500 MHz, CD_3_OD, 20 °C): *δ*=−9.21 (d, *J*=54 Hz), −11.6 ppm (d, *J*=53 Hz); HRMS (LC-ESI-TOF) calcd for C_66_H_109_N_6_O_22_P_2_^−^: 1399.7076 [*M*−H]^−^; found: 1399.7088 [*M*−H]^−^.

**Synthesis of depsi-lipid I (3)**: The phospho-Mur*N*Ac-silyl-protected-pentadepsipeptide-OTMSE **13** (12 mg, 9.5 μmol) was dissolved in dry pyridine (2.5 μL) and excess pyridine was removed under reduced pressure to give the pyridinium salt. This salt was azeotropically dried with toluene/benzene (1:1, 2×1 mL) under vacuum and the residue was dissolved in dry THF/DMF (1:1, 0.25 mL) and then added to a flask containing a solution of CDI (5.5 mg, 34 μmol) in dry THF/DMF (1:1, 0.5 mL) at 0 °C (ice/water bath) through a cannula. The whole was finally rinsed with dry THF/DMF (1:1, 3×0.2 mL). The reaction mixture was first allowed to warm to room temperature and then stirred at room temperature for 4 h at which point the reaction was complete. The reaction was then supplemented with dry methanol (3 μL), evaporated under vacuum to remove THF, and azeotropically dried with toluene/benzene (1:1, 1 mL) to yield a solution of the activated phosphoimidazolide of **13** in DMF.

The undecaprenyl phosphate diammonium salt (6.3 mg, 7.1 μmol) was azeotropically dried with toluene/benzene (1:1, 2×1 mL), and the flask was purged with argon gas. The activated phosphoimidazolide solution was then transferred to a flask containing undecaprenyl phosphate through a cannula and rinsed with dry THF/dry DMF (1:1, 2×0.2 mL). 1 *H*-Tetrazole (3.0 mg, 41 μmol) was added to the mixture and the whole was stirred at room temperature for 2.5 d. After removal of THF under reduced pressure, the residue was purified by gel permeation chromatography (Sephadex LH-20, GE Healthcare, 260×15 mm, methanol) to yield the silyl-protected depsi-lipid I (12 mg, <5.8 μmol).

The silyl-protected depsi-lipid I (12 mg) was azeotropically dried with toluene, and dissolved in dry DMF (0.3 mL). TBAF in THF (1.0 m, 0.17 mL) was then added to the protected depsi-lipid I at 0 °C, and the reaction mixture was stirred and allowed to warm to room temperature for 2 d, at which point the reaction was completed as confirmed by LC-MS. The reaction mixture was then evaporated to dryness under vacuum, and the residue was purified by gel permeation chromatography (Sephadex LH-20, GE Healthcare, 260×15 mm, methanol). The fractions containing the desired compound were combined, diluted with water, and then lyophilized overnight to yield the semi-purified depsi-lipid I as a pale brown solid (9.8 mg, <5.9 μmol). This solid was further purified by reverse-phase HPLC (Imtakt, Unison UK-C_8_, particle size 3 μm, 250×10 mm, 80 to 95 % methanol in 0.1 % NH_4_HCO_3_(aq), duration=20 min, flow rate=3 mL min^−1^, detection at *λ*=214 nm). The eluted fractions were combined and lyophilized overnight to give the depsi-lipid I (**3**) as a white solid (2.2 mg, 1.3 μmol, 23 % yield, 96 % HPLC purity). The purity of this compound was assessed by analytical method II. *R_t_*=5.6 min; [*α*]
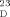
=+28° (*c*=0.10 in MeOH)); ^1^H NMR (600 MHz, CD_3_OD, 22 °C): *δ*=5.51 (dd, *J*=7.2, 3.1 Hz, 1 H), 5.45 (t, *J*=6.4 Hz, 1 H), 5.14–5.07 (comp., 10 H), 4.94 (q, *J*=7.1 Hz, 1 H), 4.52 (t, *J*=6.3 Hz, 2 H), 4.39 (q, *J*=7.3 Hz, 1 H), 4.31–4.19 (m, 4 H), 3.95 (m, 1 H), 3.86 (dd, *J*=12, 1.9 Hz, 1 H), 3.78 (dd, *J*=12, 5.5 Hz, 1 H), 3.70 (t, *J*=9.6 Hz, 1 H), 3.54 (t, *J*=9.5 Hz, 1 H), 2.91 (m, 2 H), 2.38(m, 1 H), 2.33 (m, 1 H), 2.14–2.02 (comp., 40 H), 1.99 (comp., 6 H), 1.81 (m, 1 H), 1.72 (s, 3 H), 1.66–1.65 (comp., 24 H), 1.60 (s, 3 H), 1.58 (s, 6 H), 1.47 (m, 9 H), 1.41 ppm (d, *J*=6.8 Hz, 3 H); ^13^C NMR (150 MHz, CD_3_OD, 22 °C): *δ*=175.6, 175.4, 173.9, 173.7, 140.5, 136.4, 136.3, 136.24, 136.19, 136, 135.8, 132, 126.2, 125.9, 125.53, 125.5, 125.46, 123.6, 123.5, 75.2, 72.7, 70.4, 63.7, 63.6, 55.7, 54.9, 54.8, 50.5, 49.8, 49.6, 40.9, 40.88, 40.8, 40.5, 39.9, 33.34, 33.29, 33.26, 32.9, 27.9, 27.8, 27.69, 27.66, 27.6, 27.5, 25.9, 23.9, 23.83, 23.78, 23.73, 23.6, 19.3, 18.5, 17.9, 17.8, 17, 16.2 ppm; HRMS (LC-ESI-TOF) calcd for C_86_H_143_N_6_O_22_P_2_: 1671.9608 [*M*−H]^−^; found: 1671.958 [*M*−H]^−^.

**Purification of**­ ***E. coli***­ **MurG**: *E. coli* MurG was purified as previously described by Ha et al.^35^ Briefly, *E. coli* Rosetta 2 (DE3) cells (Novagen) harboring pET21b with *murG* gene derived from *E. coli* were grown in 5 L medium supplemented with 100 μg mL^−1^ ampicillin at 37 °C. When the OD_600 nm_ reached 0.6, isopropyl β-d-1-thiogalactopyranoside (IPTG) was added to achieve a final concentration of 1 mm. The induced cell culture was grown for another 12 h at 15 °C and the cells were harvested and re-suspended in lysis buffer containing 25 mm 2-(*N*-morpholino)ethanesulfonic acid (MES) (pH 6.5), 50 mm NaCl, 1 mm dithiothreitol (DTT), 1 mm ethylenediaminetetraacetic acid (EDTA), and 2 % Triton X-100 (v/v). The cells were disrupted by a French press. The supernatant was collected at 100 000 g for 30 min at 4 °C, filtered (0.45 μm), and applied to a SP-550 column (TOYOPAERL). Bounded enzyme was eluted by using a linear gradient of NaCl from 50 to 1000 mm. The eluted fraction was dialyzed against buffer containing 50 mm Tris-HCl (pH 8.0), 500 mm NaCl, 1 mm DTT, 2 % Triton X-100 (v/v), and 5 mm imidazole. The dialyzed enzyme was then applied to a Ni^2+^ Sepharose 6 Fast Flow column (GE Healthcare) and eluted by using a linear gradient of imidazole from 5 to 300 mm. The resulting fraction was dialyzed against a buffer containing 50 mm Tris-HCl (pH 8.0), 500 mm NaCl, 1 mm DTT, and 0.5 % Triton-X (v/v). The fraction was then applied to a HiLoad 26/60 Superdex 75 column (GE Healthcare), and the collected fractions were dialyzed against a buffer containing 50 mm Tris-HCl (pH 8.0), 500 mm NaCl, 1 mm DTT, 0.5 % Triton X-100 (v/v), and 50 % glycerol (v/v). SDS-PAGE analysis of the purified protein showed an intense band at molecular weight around 40 kDa. The purity of MurG enzyme was estimated to be greater than 95 % from the Comassie blue-stained gel. The purified enzyme was stored at −80 °C until use.

**Preparation of the depsi-lipid II analogue by in vitro MurG reaction**: The C35-depsi-lipid II analogue was enzymatically synthesized by a procedure similar to that used for the preparation of the C35-lipid II by Men et al.^23^ Briefly, to a solution containing Tris-HCl (50 mm, pH 8.0), MgCl_2_ (10 mm), decyl-polyethylene glycol (0.01 %), DMSO (10 %), UDP-GlcNAc (10 mm), [^14^C]-UDP-Glc*N*Ac (0.333 mm, 300 mCi mmol^−1^), and purified *E. coli* MurG (1.1 mg mL^−1^) the C35-depsi-lipid I analogue **4** (2 μg mL^−1^) was added. The enzymatic reaction was carried out at room temperature for 60 min, and the resulting mixture was loaded onto a GL-Pak cartridge column (GL Science Inc., Tokyo, Japan) and eluted by using methanol. The resulting crude material was further purified by reverse-phase HPLC (Develosil ODS-UG-5, 4.6×150 mm, Nomura Chemical Co., Ltd., Aichi, Japan; 60 % acetonitrile/water containing 0.1 % NH_4_HCO_3_; UV detection at *λ*=210 nm). The eluate was dried under nitrogen, and then re-dissolved in Tris-HCl (50 mm, pH 8.0). The concentration of the final product (C35-depsi-lipid II analogue) was 0.328 mm (0.364 kBq μL^−1^) as ascertained by scintillation counting. The same reaction conditions were used for the preparation of the C35-lipid II analogue (0.348 mm, 0.385 kBq μL^−1^).

**Purification of**­ ***S. aureus***­ **PBP2**: The PBP2 gene was amplified from *S. aureus* RN4220 genomic DNA as a template, and the PCR product was cloned into the pET21b plasmid for expression with an N-terminal His6 fusion in *E. coli* BL21(DE3)pLysS cells. The obtained transformant was grown in 1.5 L medium supplemented with 100 μg mL^−1^ ampicillin. After incubation with shaking for 3 h at 37 °C, IPTG was added to make a final concentration of 1 mm, and the growth was continued for an additional 4 h at 25 °C. The cells were harvested and re-suspended in a solution containing Tris-HCl (20 mm, pH 8.0), NaCl (200 mm), and one tablet of Complete protease inh ibitors (Roche). After disruption of the cells by sonication, the cellular debris were collected at 75 000 g for 30 min at 4 °C, and re-suspended in a solution containing Tris-HCl (20 mm, pH 8.0), NaCl (200 mm), sodium *N*-lauroylsarcosinate, and one tablet of Complete protease inbibitors. Cells disruption was again carried out by sonication. The supernatant was collected at 75 000 g for 30 min at 4 °C and applied to a HisPrep FF Ni-NTA column (GE Healthcare). Bounded enzyme was eluted by using a linear gradient of imidazole from 20 to 500 mm. The eluted fraction was further applied to a HiLoad 26/60 Superdex 200pg column (GE Healthcare) and eluted by using a buffer containing Tris-HCl (100 mm, pH 8.0), K_2_HPO_4_ (400 mm), and DTT (1 mm). The resulting eluate was concentrated with a 10 kDa-cut off centrifugal ultrafiltration membrane, and was added to 1 vol. of glycerol. The purity of the PBP2 enzyme was estimated to be greater than 95 % from the Comassie blue-stained gel. The purified enzyme was stored at −80 °C until use.

**Initial rate measurements for the PBP2 reaction**: The enzymatic activity of the purified *S. aureus* PBP2 was evaluated by monitoring the incorporation of the [^14^C]-C35-lipid II and [^14^C]-C35-depsi-lipid II analogues into nascent peptidoglycan. The enzymatic reactions were initiated by adding each concentration of [^14^C]-C35-lipid II/[^14^C]-C35-depsi-lipid II (range from 25 to 100 μm) into the reaction mixture containing 50 mm 4-(2-hydroxyethyl)-1-piperazineethanesulfonic acid (HEPES) (pH 7.0), MnCl_2_ (18 mm), DMSO (10 %), and purified PBP2 (30 μg mL^−1^). The reaction was performed at 30 °C for 4 min and terminated by addition of 1/7 vol. of 0.25 % sodium dodecyl sulfate solution. The reaction mixture was then spotted onto a cellulose thin-layer chromatography plate (TLC plate; cellulose F precoated, Merck, Darmstadt, Germany) and developed at room temperature for 17 h with isobutyric acid/1 m ammonia (5:3) as solvent. The TLC plate was dried and then exposed to an imaging plate for 24 h in a dark room. Radioactive signals on the imaging plate were quantified with a fluorescent image analyzer (FLA3000). Data were analyzed with the MultiGuage software (GE Healthcare, Tokyo, Japan) to obtain the kinetic parameters (*V*_max_, *K*_m_, *k*_cat_) of *S. aureus* PBP2 against each substrate.

**Inhibitory effect of vancomycin and moenomycin on the reconstituted transglycosylation reaction**: The *S. aureus* PBP2 reaction and the subsequent analyses were performed as described above with minor modifications. Briefly, the reaction was initiated by adding 4 μm of [^14^C]-C35- lipid II/[^14^C]-C35-depsi-lipid II into the reaction mixture containing various concentration of each antibiotic dissolved in 10 % DMSO. The reaction was performed at 30 °C for 1 h.
