# Supplementary material for: Staphylococcus aureus Penicillin-Binding Protein 2 Can Use Depsi-Lipid II Derived from Vancomycin-Resistant Strains for Cell Wall Synthesis
Source: Chemistry. 2013 Jul 19;19(36):12104–12. doi: 10.1002/chem.201301074 (PMC4235313; doi:10.1002/chem.201301074)
Supplement: Supplementary file 1 [file chem0019-12104-SD1.pdf]

CHEMISTRY
A EUROPEAN JOURNAL

# Supporting Information

© Copyright Wiley-VCH Verlag GmbH & Co. KGaA, 69451 Weinheim, 2013

## *Staphylococcus aureus* Penicillin-Binding Protein 2 Can Use Depsi-Lipid II Derived from Vancomycin-Resistant Strains for Cell Wall Synthesis

**Jun Nakamura,[a] Hidenori Yamashiro,[b] Hiroto Miya,[a] Kenzo Nishiguchi,[b] Hideki Maki,*[b] and Hirokazu Arimoto*[a]**

chem_201301074_sm_miscellaneous_information.pdf

**Table of contents**


**Supporting Figures** **p. 2**

**Supporting Methods** **p. 4**

**Selected NMR spectra and HPLC traces of compounds** **p. 20**

**Reference** **p. 29**

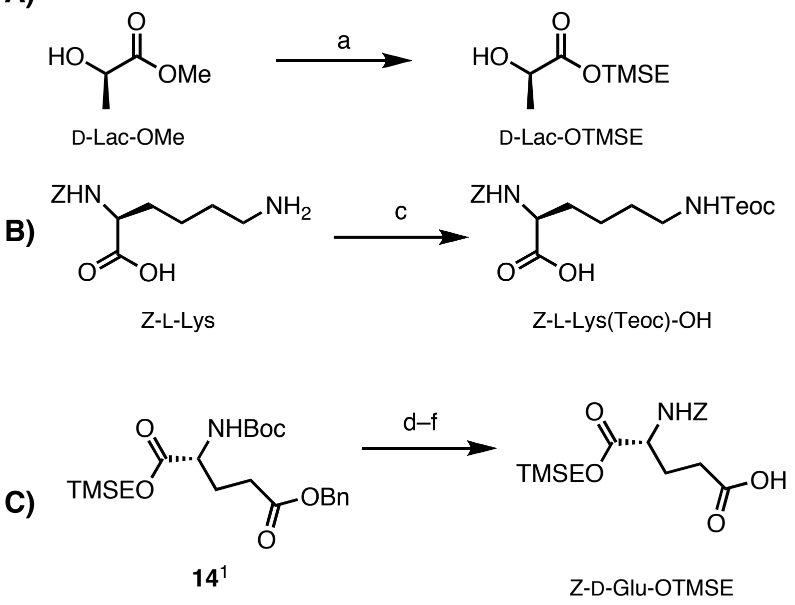


**Figure S1.** Preparation of protected lactic acid and amino acids.

*Reagents and conditions:* (a) TMSEOH, Ti(*i*-PrO)$_4$, THF, reflux, 41%; (b) Boc$_2$O, NaOH, 1,4-dioxane/water=1:1, rt, 87%; (c) Teoc-OSu, Et$_3$N, 1,4-dioxane/water=1:1, rt, 90%; (d) 10% Pd-C, H$_2$ gas, EtOH, rt, 99%; (e) TFA/CH$_2$Cl$_2$=1:4; rt, quant.; (f) Z-Cl, 2 M NaOH(aq), 0 °C, 75%.

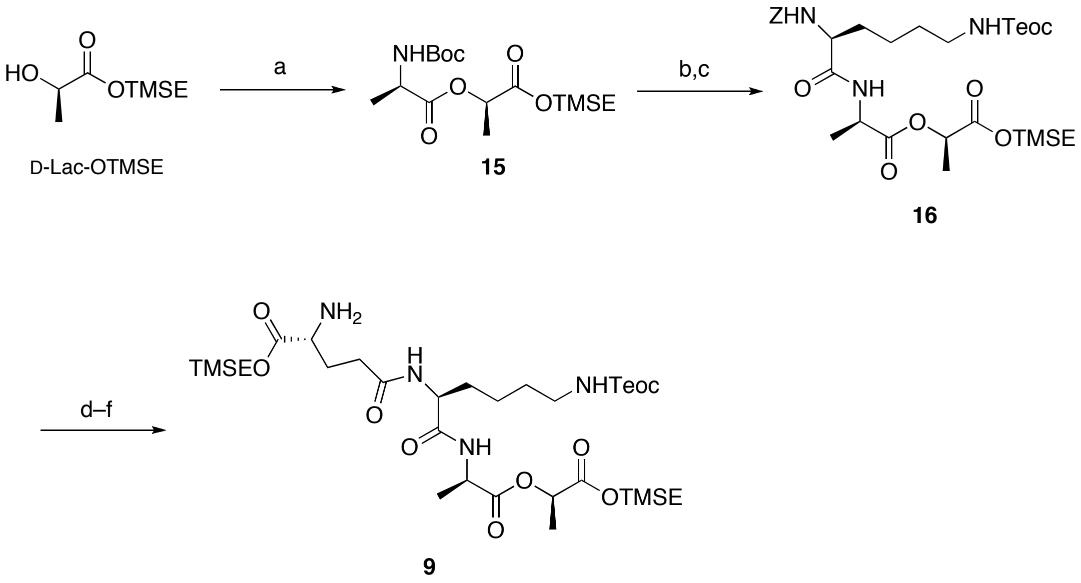


**Figure S2.** Synthesis of depsitetrapeptide.

*Reagents and conditions*: (a) Boc-D-Ala, EDC, HOBt, DMAP, CH$_2$Cl$_2$, rt, 71%; (b) TFA/CH$_2$Cl$_2$=1:1, rt, quant.; (c) Z-L-Lys(Teoc)-OH, DIPEA, PyBOP, HOBt, rt, 76%; (d) 10% Pd-C, H$_2$ gas, MeOH, rt, 90%; (e) Z-D-Glu-OTMSE, DIPEA, PyBOP, HOBt, THF/CH$_2$Cl$_2$= 1:1, rt, 75%; (f) 10% Pd-C, H$_2$ gas, MeOH, rt, 99%.

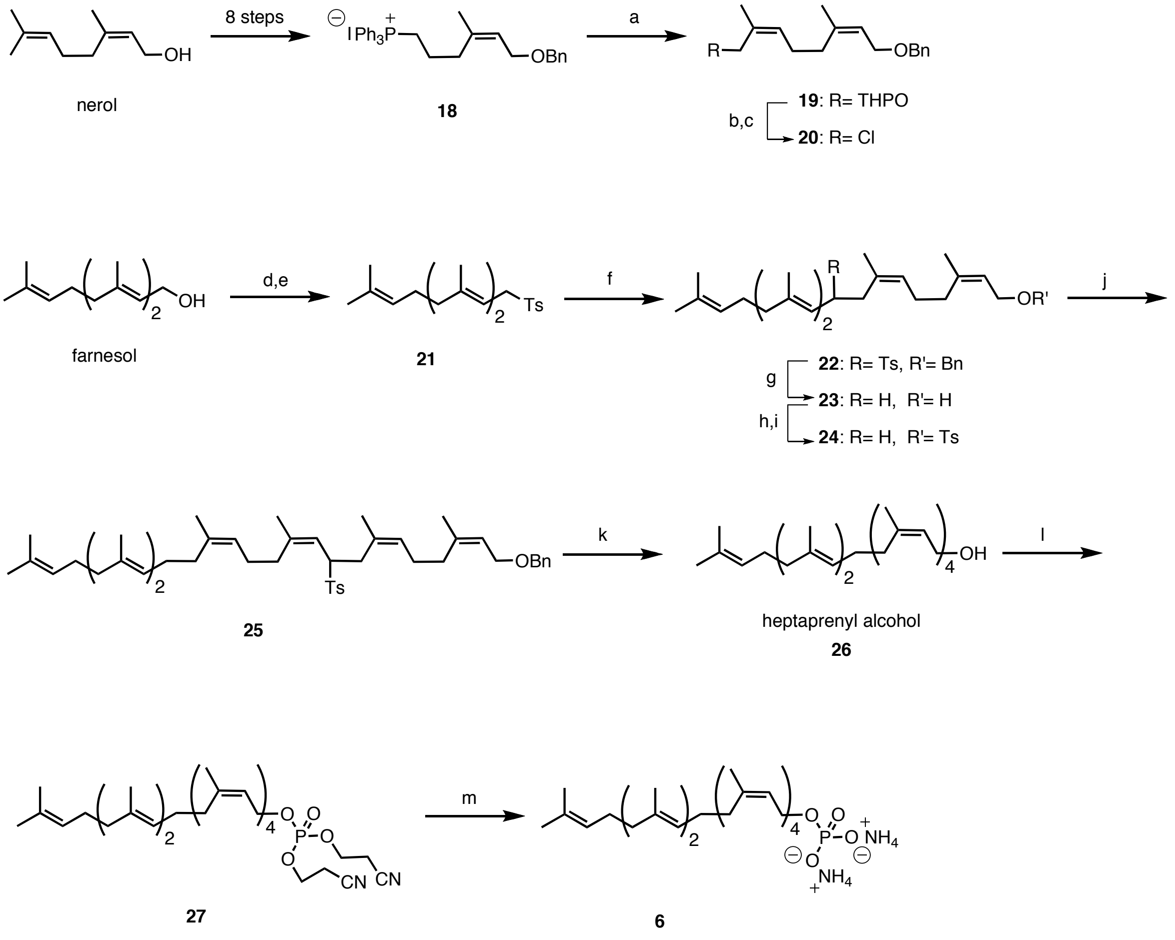


**Figure S3.** Synthesis of heptaprenylphosphate.

Reagents and conditions: (a) 1-OTHP-2-propanone, *n*-BuLi, THF, -30 ºC to -60 ºC, then warmed to rt, overnight, 60%; (b) PTSA, MeOH, rt, overnight, 85%; (c) *n*-BuLi, $Ph_3CH$, HMPA, TsCl, $Et_2O$, -60ºC, then LiCl, -60 ºC to rt, overnight, 64%; (d) *n*-BuLi, $Ph_3CH$, HMPA, TsCl, $Et_2O$, -60 ºC, then LiCl, -30 ºC to rt, overnight; (e) $NaSO_2Tol.$, DMF, 0 ºC to rt, 2 d, 56% in 2 steps from farnesol; (f) **20**, *n*-BuLi, HMPA, THF, -55 ºC, 4 h, 86%; (g) $EtNH_2/Li$, $Et_2O$, -55 ºC, 1 h, 86%; (h) *n*-BuLi, $Ph_3CH$, HMPA, TsCl, $Et_2O$, -60 ºC, then LiCl, -30 ºC to rt, overnight; (i) $NaSO_2Tol.$, DMF, 0 ºC to rt, 2 d, 60% in 2 steps from **23**; (j) **20**, *n*-BuLi, HMPA, THF, -60 ºC, 1.5 h, 89%; (k) $EtNH_2/Li$, $Et_2O$, -56 ºC, 1 h, 73%; (l) bis(2-cyanoethyl)-*N*,*N*-diisopropylphosphoramidite, 1*H*-tetrazole, THF, 0 ºC, 1 h then pyridine, $NaIO_4$, THF/water, 0 ºC, 4 h; (m) NaOMe, MeOH, rt, 2 d, 54% in 2 steps from **26**, rsm 30%.

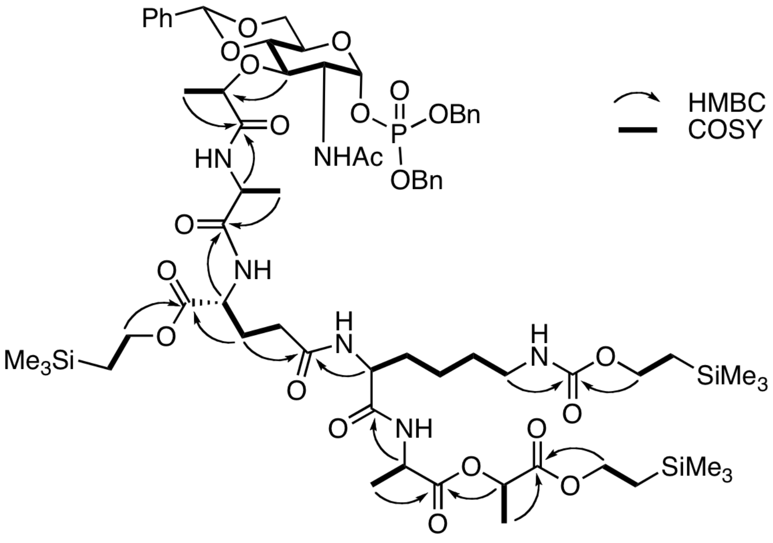


**Figure S4.** Selected COSY and HMBC correlations for **7** (600 MHz, $CD_3OD$).

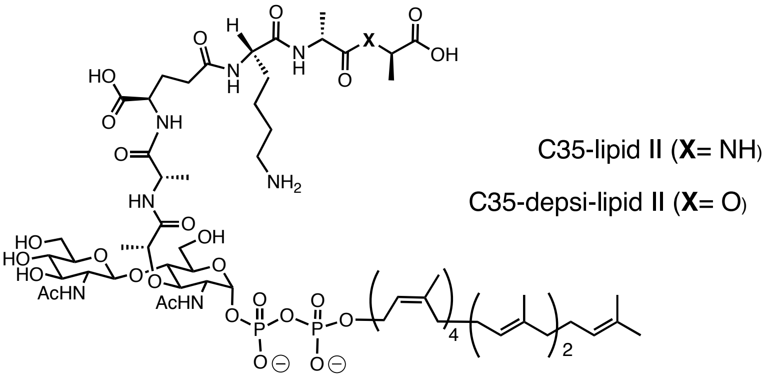


**Figure S5.** C35-lipid II and C35-*depsi*-lipid II (Glc*N*Ac is $^{14}$C-labeled).

## Supplemental methods

***Synthesis of depsitetrapeptide 9***

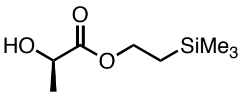


D-Lac-OTMSE

**D-Lactate TMSE ester:** A solution of (*R*)-(+)-D-lactate methyl ester (0.46 mL, 4.8 mmol) in dry THF (30 mL) was added titanium(IV) tetraisopropoxide (1.6 mL, 5.5 mmol) and 2-(trimethylsilyl)ethanol (0.82 mL, 5.7 mL). The reaction mixture was refluxed for 20 h. After the

solution was evaporated to remove THF, the residue was purified by silica gel column chromatography (*n*-hexanes/ethyl acetate=4:1), to yield D-Lac-OTMSE as a colorless oil (0.37 g, 41%). $R_f$ 0.43 (*n*-hexanes/ethyl acetate=5:1); $^1$H NMR (600 MHz, $CD_3OD$, 20 °C) δ 4.25-4.19 (comp., 3H), 1.37 (d, *J*=6.9 Hz, 1H), 1.05 (m, 2H), 0.066 (s, 9H); $^{13}$C NMR (150 MHz, $CD_3OD$, 20 °C, solvent): δ 176.5, 67.9, 64.1, 20.6, 18.2, -1.49; HRMS (FAB) calcd. for $C_8H_{19}O_3Si^+$: 190.1025 $[M+H]^+$, found 190.1024 $[M+H]^+$.

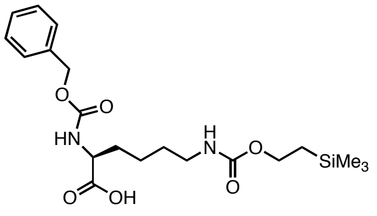


*N*-α-Z-*N*-ε-Teoc-L-Lys

***N*-α-Z-*N*-ε-Teoc-L-Lysine:** A solution of Z-L-Lys (1.0 g, 3.6 mmol) in 1,4-dioxane/water (1:1, 8 mL) was added triethylamine (0.75 mL, 5.4 mmol) and 2-(trimethylsilyl)ethyl chloroformate (Teoc-Cl, 1.0 g, 3.9 mmol). The reaction mixture was stirred at room temperature for 1 h. The reaction solution was evaporated to dryness and the residue was purified by silica gel column chromatography (chloroform/methanol=20:1), to yield *N*-Z-L-Lys(Teoc)-OH as a white solid (1.4 g, 90%). $R_f$ 0.51 (chloroform/methanol=5:1); $^1$H NMR (600 MHz, $CD_3OD$, 21 °C) δ 7.37-7.28 (comp., 5H), 5.09 (s, 2H), 4.14-4.1 (comp., 3H), 3.09 (t, *J*=6.8 Hz, 2H), 1.84 (m, 1H), 1.69 (m, 1H), 1.51 (m, 2H), 1.43 (m, 2H), 0.98 (t, *J*=8.3 Hz, 2H), 0.039 (s, 9H); $^{13}$C NMR (150 MHz, $CD_3OD$, 21 °C, solvent) δ 175.9, 159.3, 158.7, 138.2, 129.4, 128.9, 128.8, 67.6, 63.7, 55.2, 41.3, 32.4, 30.5, 24.1, 18.6, -1.44; HRMS (FAB) calcd. for $C_{20}H_{31}N_2O_6Si^-$: 423.1957 $[M-H]^-$, found 423.1952 $[M-H]^-$.

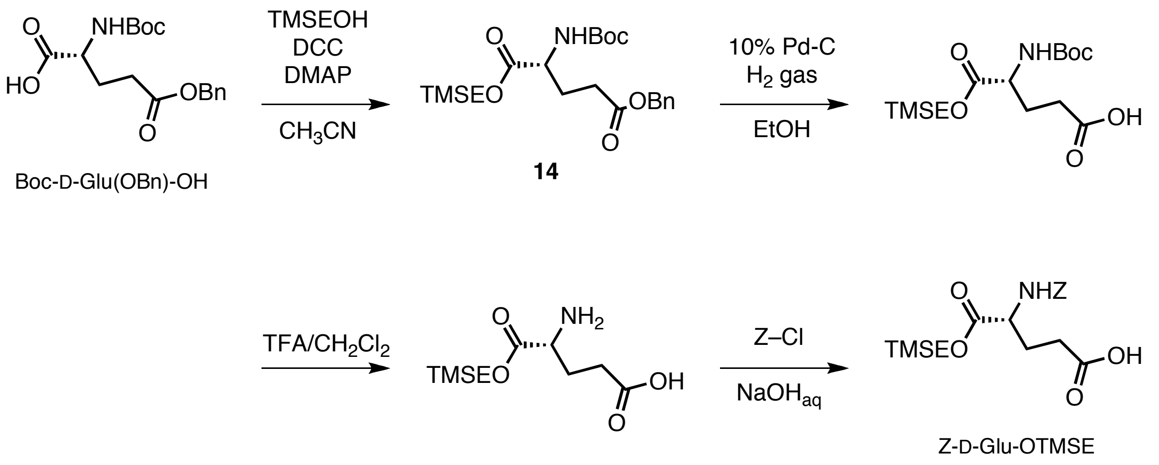

**$N$-α-Boc-γ-Benzyl-D-glutamic acid TMSE ester 14[1]:**

A solution of Boc-D-Glu(OBn)-OH (4.0 g, 12 mmol) in dry acetonitrile (95 mL) was cooled to 0 ºC, followed by addition of *N,N'*-dicyclohexylcarbodiimide (2.9 g, 14 mmol), *N,N*-dimethyl-4-aminopyridine (0.15 g, 1.2 mmol), and 2-(trimethylsilyl)ethanol (2.1 mL, 14 mL). The reaction mixture was stirred at room temperature for 8 h. The reaction solution was filtered to remove urea, and the resulting filtrate was partitioned between water (0.10 L) and ethyl acetate (3 x 0.15 L). The organic layers were combined, washed with brine, dried over anhydrous magnesium sulfate, filtered, and then evaporated to give crude **14**. Purification by silica gel column chromatography (*n*-hexane/ethyl acetate=5:1) afforded Boc-D-Glu-(OBn)-OTMSE (4.6 g, 89%).

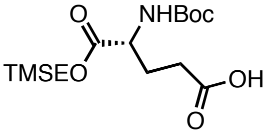


**$N$-α-Boc-D-Glutamic acid TMSE ester:** 10% Pd/C catalyzed hydrogenation of Boc-D-Glu-(OBn)-OTMSE (4.2 g, 9.6 mmol) in ethanol (50 mL) at room temperature for 2 h afforded Boc-D-Glu-OTMSE as a colorless solid (3.3 g, 99%), which was used for the next reaction without further purification.

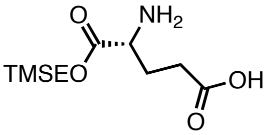


**D-Glutamic acid TMSE ester:** Boc-D-Glu-OTMSE (2.3 g, 6.6 mmol) was dissolved in dichloromethane/TFA (4:1, 50 mL), and stirred at room temperature for 1 h. The reaction mixture was diluted with toluene (0.10 L) and evaporated to dryness. The residue was purified by silica gel column chromatography (chloroform/methanol=8:1) to yield D-Glu-OTMSE (1.6 g, 98%).

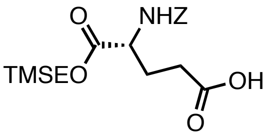


**$N$-Z-D-Glutamic acid TMSE ester:**

D-Glu-OTMSE (1.6 g, 6.6 mmol) was dissolved in 2 M $NaOH_{aq}$ (7.4 mL) at 0 ºC, and added benzyl chloroformate (1.5 g, 8.6 mmol). The reaction mixture was stirred at 0 ºC for 1 h, and then added 1

M $HCl_{aq}$ to adjust the pH to 2. The reaction solution was partitioned between brine (20 mL) and an ethyl acetate (3 x 30 mL). The organic layers were combined, washed with brine, dried over anhydrous magnesium sulfate, filtered, and evaporated to give a crude residue. This residue was purified by silica gel column chromatography (*n*-hexane/ethyl acetate=1:1) to afford Z-D-Glu-OTMSE (2.5 g, 75%). $R_f$ 0.56 (chloroform/methanol=10:1); $^1$H NMR (600 MHz, $CD_3OD$, 21 °C) δ 7.39-7.27 (comp., 5H), 5.11 (d, *J*=12 Hz, 1H), 5.08 (d, *J*=12 Hz, 1H), 4.23-4.2 (d, *J*=12 Hz, 1H), 2.41 (t, *J*=7.3 Hz, 2H), 2.15 (m, 1H), 1.95 (m, 1H), 1.03 (m, 2H), 0.051 (s, 9H); $^{13}$C NMR (150 MHz, $CD_3OD$, 21 °C, solvent) δ 176.1, 173.7, 158.5, 138, 129.4, 128.9, 128.7, 67.6, 64.6, 54.9, 31, 27.7, 18.1, -1.46; HRMS (FAB) calcd. for $C_{18}H_{26}NO_6Si^-$: 380.1535 $[M–H]^-$, found 380.1533 $[M–H]^-$.

***N*-α-Boc-D-Alanyl-D-lactate TMSE ester 15:**

A mixture of D-Lac-OTMSE (2.5 g, 13 mmol), Boc-D-Ala (2.5 g, 13 mmol), 1-hydroxybenzotriazole (HOBt, 1.8 g, 13 mmol), and DMAP (0.16 g, 1.3 mmol) were treated with 1-ethyl-3-(3-dimethylaminopropyl)carbodiimide (EDC, 3.3 g, 17 mmol) at 0 °C to room temperature overnight. The reaction solution was partitioned between brine (50 mL) and ethyl acetate (3 x 60 mL). The organic layers were combined, washed with brine, filtered, and evaporated to give crude dipeptide (6.7 g). Purification by silica gel column chromatography (*n*-hexane/ ethyl acetate=5:1) afforded Boc-D-Ala-D-Lac-OTMSE (3.4 g, 71%). $R_f$ 0.82 (chloroform/methanol/acetic acid=95:5:3); $^1$H NMR (600 MHz, $CD_3OD$, 21 °C) δ 5.06 (q, *J*=7.4 Hz, 1H), 4.25 (m, 2H), 4.18 (q, *J*=7.4 Hz, 1H), 1.51 (d, *J*=7.2 Hz, 3H), 1.49 (d, *J*=7.4 Hz, 3H), 1.44 (s, 9H), 1.4 (d, *J*=7.4 Hz, 3H), 1.03 (t, *J*=8.2 Hz, 2H) 0.063 (s, 9H); $^{13}$C NMR (150 MHz, $CD_3OD$, 21 °C) δ 174.4, 172.2, 157.9, 80.5, 70.7, 64.7, 50.4, 28.7, 18.2, 17.5, 17.1, -1.54; HRMS (FAB) calcd. for $C_{16}H_{32}NO_6Si^+$: 362.1999 $[M+H]^+$, found 362.2001 $[M+H]^+$.

***N*-α-Z-*N*-ε-Teoc-L-Lysyl-D-alanyl-D-lactate TMSE ester 16:**

Boc-D-Ala-D-Lac-OTMSE (0.94 g, 2.6 mmol) was dissolved in dichloromethane/TFA (1:1, 5 mL) mixture, and the mixture was stirred at room temperature for 30 min. The reaction was diluted with toluene (2 mL) and evaporated to give a white solid containing D-Ala-D-Lac-OTMSE (0.68 g, 99%). This material was used for the next reaction without further purification.

A solution of D-Ala-D-Lac-OTMSE (0.64 g, 2.5 mmol) in dry THF/dichloromethane (1:1, 25 mL) was added *N*-Z-L-Lys(Teoc)-OH (1.1 g, 2.6 mmol), DIPEA (0.9 mL, 5.1 mmol), PyBOP (1.5 g, 2.8 mmol) and HOBt (0.35 g, 2.6 mmol) at room temperature. The reaction mixture was stirred at

room temperature for 1 h, at which point the starting material was completely consumed. The reaction solution was partitioned between water (0.10 L) and ethyl acetate (5 x 0.10 L). The organic layers were combined, washed with brine, dried over anhydrous magnesium sulfate, filtered, and evaporated to give a crude oil. Purification by silica gel column chromatography (*n*-hexane/ethyl acetate=2:1) afforded Z-L-Lys(Teoc)-D-Ala-D-Lac-OTMSE as a colorless solid (1.1 g, 67%). $R_f$ 0.42 (chloroform/methanol=20:1); $^1$H NMR (600 MHz, $CD_3OD$, 20 °C) δ 7.36-7.27 (comp., 5H), 5.09 (s, 2H), 5.06 (q, *J*=7.3 Hz, 1H), 4.47 (q, *J*=6.8 Hz, 1H), 4.26 (m, 2H), 4.15 (m, 1H), 4.12 (t, *J*=8.5 Hz, 2H), 3.08 (t, *J*=6.4 Hz, 2H), 1.77 (m, 1H), 1.65 (m, 1H), 1.49 (m, 2H), 1.48 (d, *J*=7.4 Hz, 3H), 1.44 (d, *J*=7.3 Hz, 3H), 1.38 (m, 2H), 1.02 (t, *J*=8.3 Hz, 2H), 0.98 (t, *J*=8.3 Hz, 2H), 0.056 (s, 9H), 0.04 (s, 9H); $^{13}$C NMR (150 MHz, $CD_3OD$, 20 °C) δ 175.9, 174.5, 173.2, 160.4, 159.5, 139.4, 130.7, 130.2, 130.1, 80.7, 71.8, 68.9, 66, 64.9, 57.5, 42.5, 34.3, 31.7, 25.1, 19.9, 19.4, 18.6, 18.4, -0.133, -0.219; HRMS (FAB) calcd. for $C_{31}H_{54}N_3O_9Si_2^+$: 668.3399 $[M+H]^+$, found 668.3400 $[M+H]^+$.

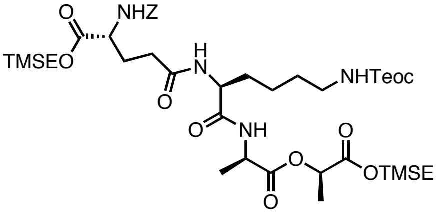


***N*-Z-α-OTMSE-γ-D-Glutamyl-*N*-ε-Teoc-L-lysyl-D-alanyl-D-lactate TMSE ester:**

10% Pd/C catalyzed hydrogenation of Z-L-Lys(Teoc)-D-Ala-D-Lac-OTMSE (0.12 g, 0.18 mmol) in methanol (2 mL) at room temperature for 30 min afforded L-Lys(Teoc)-D-Ala-D-Lac-OTMSE as a colorless solid (86 mg, 90%). This material was used for the next reaction without further purification.

L-Lys(Teoc)-D-Ala-D-Lac-OTMSE (0.49 g, 0.92 mmol) in dry THF/dichloromethane (1:1, 10 mL) was added Z-D-Glu-OTMSE (0.37 g, 0.96 mmol), DIPEA (0.33 mL, 1.9 mmol), PyBOP (0.55 g, 1.1 mmol) and HOBt (0.13 g, 0.95 mmol). The reaction mixture was stirred at room temperature for 8 h. The reaction solution was then partitioned between water (0.10 L) and ethyl acetate (3 x 0.10 L). The organic layers were combined, washed with brine, dried over anhydrous magnesium sulfate, filtered, evaporated to give a crude oil. Purification by silica gel column chromatography (*n*-hexane/ethyl acetate=1:1) afforded Z-γ-D-Glu(OTMSE)-L-Lys(Teoc)-D-Ala-D-Lac-OTMSE as a white solid (0.62 g, 75%). $R_f$ 0.38 (chloroform/methanol=20:1); $^1$H NMR (600 MHz, $CD_3OD$, 21 °C) δ 7.38-7.28 (comp., 5H), 5.13 (d, *J*=12 Hz, 1H), 5.08 (d, *J*=12 Hz, 1H), 5.05 (q, *J*=7.4 Hz,

1H), 4.49 (q, *J*=7.3 Hz, 1H), 4.34 (dd, *J*=8.5, 5.8 Hz, 1H), 4.23-4.17 (comp., 5H), 4.12 (t, *J*=8.2 Hz, 2H), 3.1 (t, *J*=6.9 Hz, 2H), 2.39 (m, 2H), 2.07 (m, 1H), 1.85 (m, 1H), 1.78 (m, 1H), 1.65 (m, 1H), 1.51 (m, 2H), 1,49 (d, *J*=6.9 Hz, 3H), 1.45 (d, *J*=7.3 Hz, 3H), 1.39 (m, 2H), 1.06-0.96 (comp., 6H), 0.066-0.051 (comp., 27H)); $^{13}$C NMR (150 MHz, $CD_3OD$, 21 °C) δ 174.7, 174.2, 173.6, 173.4, 172, 159.2, 158.5, 138.1, 129.5, 129, 128.9, 79.4, 70.5, 67.8, 64.7, 64.6, 63.6, 54.8, 54.6, 41.4, 41.2, 32.6, 30.5, 28.6, 23.9, 18.6, 18.2, 18.1, 17.3, 17.1, -1.37, -1.42, -1.46; HRMS (FAB) calcd. for $C_{41}H_{72}N_4NaO_{12}Si_3^+$: 919.4352 $[M+Na]^+$, found 919.4357 $[M+Na]^+$.

**α-OTMSE-γ-D-Glutamyl-*N*-ε-Teoc-L-lysyl-D-alanyl-D-lactate TMSE ester 9:**

10% Pd/C catalyzed hydrogenation of Z-γ-D-Glu(OTMSE)-L-Lys(Teoc)-D-Ala-D-Lac-OTMSE (0.43 g, 0.47 mmol) in methanol (5 mL) at room temperature for 2 h afforded γ-D-Glu(OTMSE)-L-Lys(Teoc)-D-Ala-D-Lac-OTMSE as a colorless solid (0.36 g, 99%). This material was used for the next reaction without further purification.

Purification of crude γ-D-Glu(OTMSE)-L-Lys(Teoc)-D-Ala-D-Lac-OTMSE (29 mg) by silica gel column chromatography (chloroform/methanol=10:1) gave **9** as a colorless oil (23 mg, 30 μmol), which provided the following compound data. $R_f$ 0.40 (chloroform/methanol=10:1); $^1$H NMR (600 MHz, $CD_3OD$, 20 °C) δ 5.06 (q, *J*=6.9 Hz, 1H), 4.44 (q, *J*=7.1 Hz, 1H), 4.36 (dd, *J*=8.3, 5.5 Hz, 1H), 4.25-4.19 (comp., 4H), 4.13 (t, *J*=8.3 Hz, 2H), 3.44 (t, *J*=7.3 Hz, 1H), 3.09 (t, *J*=6.9 Hz, 2H), 2.41 (m, 1H), 2.25 (m 1H), 1.89 (m, 1H), 1.8 (m, 1H), 1.67 (m, 1H), 1.48 (m, 2H), 1.47 (d, *J*=6.8 Hz, 3H), 1.46 (d, *J*=7.3 Hz, 3H), 1.41 (m, 2H), 1.05 (m, 6H), 0.054 (s, 9H), 0.043 (s, 9H), 0.04 (s, 9H); $^{13}$C NMR (150 MHz, $CD_3OD$, 20 °C) δ 175.6, 172.9, 172.2, 171.4, 170.4, 157, 69.2, 63.9, 63.4, 62.9, 53.7, 52.6, 48, 40.1, 32.9, 31.1, 29.7, 29.6, 22.4, 17.9, 17.7, 17.4, 17.3, 16.8, -1.48, -1.54, -1.56; HRMS (FAB) calcd. for $C_{33}H_{67}N_4O_{10}Si_3^+$: 763.4165 $[M+H]^+$, found 763.4163 $[M+H]^+$

***Synthesis of heptaprenyl phosphate diammonium salt 6***

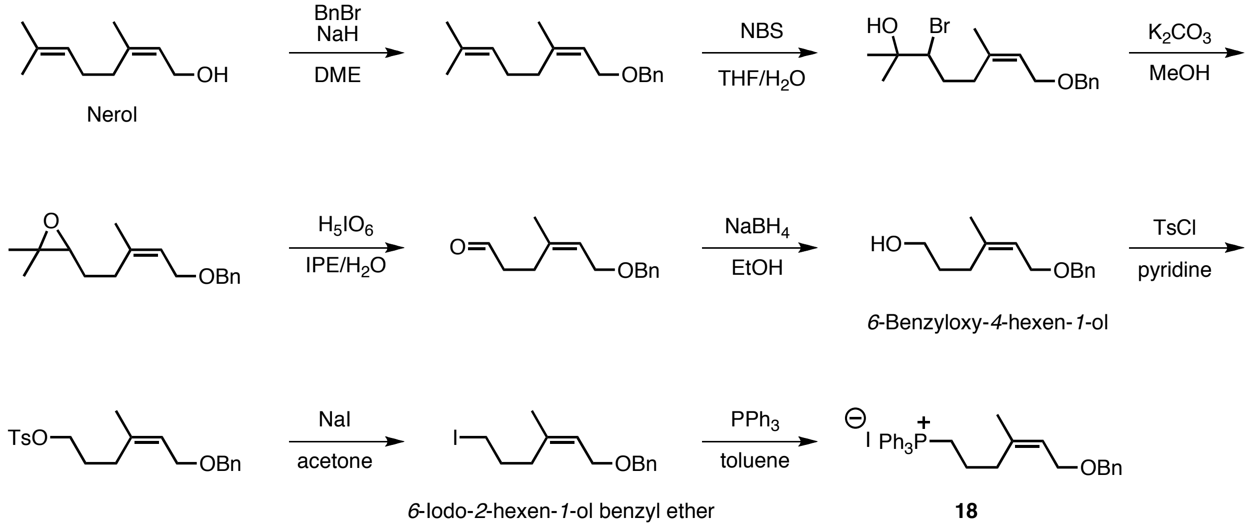


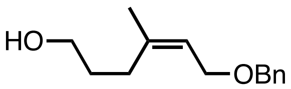


***6*-Benzyloxy-*4*-methyl-*4Z*-hexen-*1*-ol:**

Sodium hydride (60-72% in mineral oil, 72 g, 1.8 mol) was washed with *n*-hexane, dried under a vacuum and then suspended in dimethoxyethane (DME, 1.5 L). This suspension was added a solution of nerol (0.23 kg, 1.5 mol) in DME (1.5 L) over 60 min. The reaction mixture was heated to 75 ºC and stirred for 2 h. After cooling to room temperature, the mixture was added benzyl bromide (0.20 L, 1.6 mol), and then refluxed for 1 h, at which point the starting material was completely consumed. The reaction mixture was poured into ice-cold brine (2.5 L) and then extracted with *n*-hexane (5 x 1.0 L). The organic layers were combined, evaporated, washed with brine, dried over anhydrous magnesium sulfate, filtered and concentrated under reduced pressure to give a yellow liquid containing nerol benzyl ether (0.38 kg). This material was used for the next sample without further purification. $R_f$ 0.73 (*n*-hexane/ethyl acetate=2:1).

A solution of the crude nerol benzyl ether (0.38 kg) in THF (3.0 L) was added water (0.70 L). After being cooled to 0 ºC, *N*-bromo succinimide (0.33 kg) was added over 2 h, at which point the starting material was consumed. The reaction was added brine (1.0 L) and extracted with *i*-propyl ether (3 x 1.0 L). The organic layers were combined, washed with brine, dried over anhydrous magnesium sulfate, filtered and concentrated to give a yellow liquid containing *6,7*-bromohydrine-*3,7*-dimethyl-*2Z*-octen-*1*-ol benzyl ether (0.57 kg). This material was used for

the next reaction without further purification. $R_f$ 0.48 (*n*-hexane/ethyl acetate=2:1).

A solution of bromohydrine (0.57 kg) in methanol (3.0 L) was cooled down to -5 ºC and treated with potassium carbonate (0.31 kg). The reaction mixture was stirred at -5 ºC for 1 h. The reaction was diluted with brine (2.0 L) and extracted with *i*-propyl ether (2 x 2.0 L). The organic layers were combined, washed with brine, dried over anhydrous magnesium sulfate, filtered, and concentrated under reduced pressure to give a yellow liquid containing *6,7*-epoxy-*3,7*-dimethyl-*2Z*-octen-*1*-ol benzyl ether (0.48 kg). This material was used for the next reaction without further purification. $R_f$ 0.55 (*n*-hexane/ethyl acetate=2:1).

A solution of the crude epoxide (0.48 kg) in *i*-propyl ether (2.5 L) was cooled to -10 ºC and then added 37% (w/w) *ortho*-periodic acid in water (0.82 L). The reaction was stirred and allowed to warm to room temperature for 14 h at which point the starting material was consumed. The reaction mixture was partitioned with brine (2.0 L) and the organic layer was separated. The organic layer was washed with brine, dried over anhydrous magnesium sulfate, filtered, evaporated to give a red liquid containing *6*-benzyloxy-*4*-methyl-*4Z*-hexenal (0.45 kg). This sample was used for the next reaction without further purification. $R_f$ 0.36 (*n*-hexane/ethyl acetate=4:1).

*6*-Benzyloxy-*4*-hexenal (0.45 kg) was dissolved in ethanol (1.5 L) and cooled to -10 ºC. The solution was added sodium borohydride (28 g, 0.75 mmol) and stirred for 1 h. Additional portion of sodium borohydride (2.8 g, 75 mmol) was added to complete the reaction and then reaction mixture was stirred at room temperature for 30 min, at which point the aldehyde was consumed. The reaction mixture was added 1M $HCl_{aq}$ (0.65 L) in a dropwise manner and then added water (0.80 L), and extracted with ethyl acetate (4 x 1.5 L). The organic layers were combined, washed with brine, dried over anhydrous magnesium sulfate, filtered and evaporated to give an orange liquid containing *6*-benzyloxy-*4*-methyl-*4Z*-hexen-*1*-ol (0.41 g). Purification by silica gel column chromatography afforded the title compound (84 g, 25% in five steps from nerol). $R_f$ 0.50 (*n*-hexane/ethyl acetate=1:1); $^{1}$H NMR (500 MHz, $CDCl_3$, 20 °C) δ 7.37-7.27 (comp., 5H), 5.52 (t, *J*=7 Hz, 1H), 4.52 (s, 2H), 4 (d, *J*=7.5 Hz, 2H), 3.56 (t, *J*=6 Hz, 2H), 2.33 (s, 1H), 2.2 (t, *J*=7.5 Hz 2H), 1.75 (s, 3H), 1.69 (quin., *J*=7 Hz, 2H); $^{13}$C NMR (125 MHz, $CDCl_3$, 20 °C) δ 141.6, 138, 128.4, 127.9, 127.7, 121.7, 72.4, 65.7, 61.2, 30.1, 27.7, 23.1; HRMS (FAB) calcd. for $C_{14}H_{21}O_2^+$: 221.1542 $[M+H]^+$, found 221.1542 $[M+H]^+$.

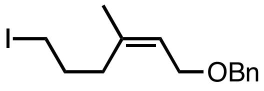


**6-Iodo-3-methyl-2Z-hexen-1-ol benzyl ether 17:**

*6*-Benzyloxy-*4*-hexenol (10 g, 45 mmol) was dissolved in pyridine (90 mL), and then cooled to -5 ºC. The solution was added *p*-toluenesulfonyl chloride (17 g, 91 mmol) and stirred at -5 ºC for 8 h. The reaction mixture was poured into a mixture of ethyl acetate/ice-water (1:1, 0.80 L) and partitioned. The organic layer was washed successively with 10% aq. $CuSO_4$ (0.40 L), water and brine. The solution was dried over anhydrous magnesium sulfate, filtered, concentrated to give an orange liquid containing *6*-benzyloxy-*4*-methyl-*4Z*-hexen-*1*ol *p*-toluenesulfonate (14 g). $R_f$ 0.51 (*n*-hexane/ethyl acetate=5:1). This material was used for the next reaction without further purification.

The crude *6*-benzyloxy-*4*-hexenol *p*-toluenesulfonate (14 g) was added to a solution of sodium iodide (16 g, 0.11 mol) in dry acetone (40 mL) in a dropwise manner at room temperature. The reaction mixture was stirred at room temperature for 41 h at which point the starting material was completely consumed. The reaction solution was poured into 0.03 M sodium thiosulfate$_{aq}$/*n*-hexane (1:1, 0.80 L) and extracted with *n*-hexane (2 x 0.30 L). The organic layers were combined, washed with brine, dried over anhydrous magnesium sulfate, filtered, and evaporated to give the title compound as a yellow liquid (10 g). Purification by silica gel column chromatography (*n*-hexane/ethyl acetate=20:1) afforded *6*-iodo-*2*-hexenol benzyl ether as a colorless liquid (8.3 g, 69% in 2 steps from *6*-benzyloxy-*4*-hexenol). $R_f$ 0.58 (*n*-hexane/ethyl acetate=10:1); $^1$H NMR (500 MHz, $CDCl_3$, 20 °C) δ 735-7.27 (comp., 5H), 5.49 (t, *J*=7 Hz, 1H), 4.52 (s, 2H), 4.04 (d, *J*=7 Hz, 2H), 3.14 (t, *J*=7 Hz, 2H), 2.16 (t, *J*=7 Hz, 2H), 1.75 (s, 3H); $^{13}$C NMR (125 MHz, $CDCl_3$, 20 °C) δ 138.8, 138.3, 128.4, 127.9, 127.6, 123, 72.3, 66.3, 32.7, 31.8, 23.4, 6.35; HRMS (EI) calcd. for $C_{14}H_{19}IO^+$: 330.0481 $[M]^+$, found 331.0488 $[M]^+$.

***6*-Benzyloxy-*4*-methyl-*4Z*-hexen-*1*-yl-triphenyl phosphonium iodide 18:** A solution of *6*-iodo-*2*-hexenol benzyl ether (**17**, 0.91 g, 2.8 mmol) in dry toluene (7 mL) was added triphenylphosphine (5.4 g, 21 mmol). The reaction mixture was refluxed for 117 h at which point the starting material was consumed. Cooling of the reaction to room temperature resulted in generation of white precipitate. The precipitate was decanted, and recrystallized from toluene to yield a white solid (1.6 g, 97%). $R_f$ 0.01 (*n*-hexane/*i*-propyl ether=5:1); $^1$H NMR (500 MHz, $CDCl_3$, 20 °C) δ 7.8-7.76 (comp., 9H), 7.69-7.63 (comp., 6H), 7.33-7.23 (comp., 5H), 5.54 (t, *J*=6.5 Hz, 1H), 4.57 (s, 2H), 4.1 (d, *J*=7 Hz, 2H), 3.78 (m, 2H), 2.53 (t, *J*=7 Hz, 2H), 1.81 (m, 2H), 1.6 (s, 3H), 1.58 (s, 3H); $^{13}$C NMR (125 MHz, $CDCl_3$, 20 °C) δ 138.5, 138.4, 135.03, 135.00, 133.6, 133.5, 130.5, 130.4, 128.3, 127.9, 127.4, 124.3, 118.4, 117.8, 72.6, 66.5, 32.1, 23.1, 22, 20.3; HRMS (FAB) calcd. for $C_{32}H_{34}OP^+$: 465.2342 $[M]^+$, found 465.2347 $[M]^+$.

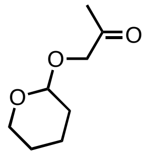

Hydroxyacetone THP ether

**Hydroxyacetone *1*-(tetrahydro-2*H*-pyran-*2*-yl) ether:**

A mixture of hydroxyacetone (50 g, 0.68 mol) and *3,4*-dihydro-2*H*-pyrane (59 g, 0.68 mol) was added pyridinium *p*-toluenesulfonate (17 g, 68 mmol) and then stirred at room temperature for 27 h. The reaction mixture was concentrated under reduced pressure to remove THF, and poured into ethyl acetate (0.50 L) and then partitioned between brine (0.50 L) and ethyl acetate (2 x 0.50 L). The organic layers were combined, dried over anhydrous magnesium sulfate, filtered, evaporated to give a whitish liquid (92 g). Purification of this sample by silica gel column chromatography (*n*-hexane/ethyl acetate=2:1) afforded the title compound as a colorless liquid (55 g, 51%). $R_f$ 0.40 (*n*-hexane/ethyl acetate=1:1); $^{1}$H NMR (500 MHz, $CDCl_3$, 20 °C): δ 4.66 (t, *J*=3.5 Hz, 1H), 4.28 (d, *J*=18 Hz, 1H), 4.14 (d, *J*=18 Hz, 1H), 3.87 (m, 1H), 3.54 (m, 1H), 2.19 (s, 3H), 1.89-1.54 (comp., 6H); $^{13}$C NMR (125 MHz, $CDCl_3$, 20 °C) δ 206.7, 98.7, 72.3, 62.3, 30.2, 26.5, 25.2, 19.1; HRMS (FAB) calcd. for $C_8H_{15}O_3^+$: 159.1021 $[M+H]^+$, found 159.1029 $[M+H]^+$.

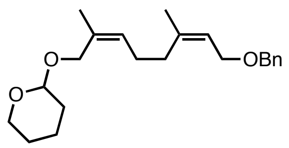

*8*-Benzyloxy-*2,6*-dimethylocta-*2,6*-dien-*1*-ol *2*-THP acetal

***8*-Benzyloxy-*2,6*-dimethylocta-*2Z,6Z*-dien-*1*-ol *2*-tetrahydro-2*H*-furan acetal 19:**

*6*-Benzyloxy-*4*-hexenyl-triphenyl phosphonium iodide **18** (30 g, 51 mmol) was azeotropically dried with toluene (30 mL) and placed under a vacuum. This sample was dissolved in dry THF (3 L) and added *n*-butyl lithium (1.59 M solution in *n*-hexane, 37 mL, 61 mmol) over 6 min at -12 ºC . A solution of hydroxyacetone THP ether (8 g, 51 mmol) in dry THF (20 mL) was added to the reaction solution and rinsed with dry THF (2 x 10 mL). The reaction mixture was stirred and allowed to warm to room temperature overnight. After filtration and removal of solvent, the resulting residue was dissolved in *n*-hexane/*i*-propyl ether (1:1, 0.70 L) and then partitioned between a water layer (water, 0.70 L) and an organic layer (*n*-hexane/*i*-propyl ether=1:1, 4 x 1 L). The organic layers were concentrated and then combined, washed with brine, dried over anhydrous magnesium sulfate, filtered and then concentrated under reduced pressure to give a heterogeneous mixture of a white solid and an orange liquid (18 g). Purification of this mixture by silica gel

column chromatography (*n*-hexane/ethyl acetate=20:1) afforded *8*-benzyloxy-octadien-*1*-ol *2*-THP acetal **19** as a colorless oil (11 g, 60%). $R_f$ 0.49 (*n*-hexane/ethyl acetate=5:1), $^{1}$H NMR (500 MHz, $CDCl_3$, 20 °C, tetramethylsilane) δ 7.34-7.27 (comp., 5H), 5.43 (t, *J*=6 Hz, 1H), 5.34 (t, *J*=7 Hz, 1H), 4.58 (t, *J*=3 Hz, 1H), 4.49 (s, 2H), 4.11 (t, *J*=12 Hz, 2H), 4.01 (d, *J*=6.5 Hz, 2H), 3.89 (dt, *J*=9, 9, 3 Hz, 1H), 3.51 (m, 1H), 2.17-2.06 (comp., 4H), 1.85 (m, 1H), 1.75 (s, 6H), 1.7 (m, 1H), 1.59-1.52 (comp., 4H); $^{13}$C NMR (125 MHz, $CDCl_3$, 20 °C, solvent) δ 140.1, 138.5, 132.3, 128.7, 128.3, 127.8, 127.5, 122.2, 97.4, 72.1, 66.3, 65.2, 62.1, 32.4, 30.6, 26.2, 25.5, 23.4, 21.7, 19.5; HRMS (FAB) calcd. for $C_{22}H_{33}O_3^+$: 345.2430 $[M+H]^+$, found 345.2438 $[M+H]^+$.

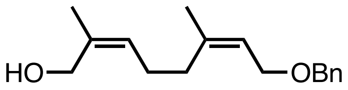


*8*-Benzyloxy-*2,6*-dimethylocta-*2,6*-dien-*1*-ol

**8-Benzyloxy-2,6-dimethylocta-2Z,6Z-dien-1-ol:**

A solution of *8*-benzyloxy-octadien-*1*-ol *2*-THP acetal **19** (2.2 g, 6.2 mmol) in methanol (72 mL), was added *p*-toluenesulfonic acid monohydrate (0.12 g, 0.62 mmol). The reaction mixture was stirred at room temperature for 18 h. The reaction solution was poured into a mixture of brine/*i*-propyl ether (1:1, 1.0 L) and partitioned, dried over anhydrous magnesium sulfate, filtered, evaporated to give a pale yellow liquid (2.1 g). Purification of this material by silica gel column chromatography (*n*-hexane/ethyl acetate=5:1) afforded the title compound as a colorless liquid (1.4 g, 86%). $R_f$ 0.17 (*n*-hexane/ethyl acetate=5:1); $^{1}$H NMR (500 MHz, $CDCl_3$, 20 °C) δ 7.36-7.27(comp., 5H), 5.46 (t, *J*=7 Hz, 1H), 5.26 (t, *J*=7 Hz, 1H), 4.49 (s, 2H), 4.04 (s, 2H), 2.97 (d, *J*=7 Hz, 2H), 2.15-2.06 (comp., 4H), 1.77 (s, 3H), 1.76 (s, 3H), 1.72 (s, 1H); $^{13}$C NMR (125 MHz, $CDCl_3$, 20 °C) δ 140.3, 138.4, 135.1, 128.3, 127.8, 127.5, 127.4, 122.1, 72.2, 66.2, 61.3, 32.3, 26.1, 23.6, 21.3; HRMS (FAB) calcd. for $C_{17}H_{25}O_2^+$: 261.1855 $[M+Na]^+$, found 261.1856 $[M+Na]^+$.

**8-Chloro-3,7-dimethylocta-2Z,6Z-dien-1-ol benzyl ether 20:**

A solution of *8*-benzyloxy-dienol (24 g, 91 mmol) in dry diethyl ether (140 mL) and HMPA (60 mL) was added triphenylmethane (2.3 g, 9.7 mmol) at room temperature. After being cooled to -55 ºC, the mixture was added *n*-butyl lithium (1.59 M solution in THF, 72 mL, 0.12 mol) in a dropwise manner over 30 min, and then added a solution of *p*-toluenesulfonyl chloride (TsCl, 26 g, 0.12 mol) in dry diethyl ether (90 mL) and rinsed with dry diethyl ether (5 x 4 mL). To complete the reaction, the mixture was added additional TsCl (8.1 g, 42 mmol) in dry diethyl ether (40 mL) and stirred for another 2 h. The reaction was added lithium chloride (5.7 g, 0.13 mol) at -30 ºC and

then warmed to room temperature for 14 h. The reaction solution was poured into water (0.70 L) and partitioned between a water layer and an organic layer (*i*-propyl ether, 2 x 0.70 L). The organic layers were combined, washed with brine, dried over anhydrous magnesium sulfate, filtered, evaporated to give a crude residue containing **20** as a yellow liquid (40 g). Purification of this material by silica gel column chromatography (*n*-hexane/ethyl acetate=100:1) gave a pale yellow liquid (23 g), and further purification (silica gel column chromatography, *n*-hexane/ethyl acetate=25:1) afforded *8*-chlorodiene-*1*-ol benzyl ether **20** as colorless oil (16 g, 84%). $R_f$ 0.71 (*n*-hexane/ethyl acetate=5:1, double development); $^1$H NMR (500 MHz, $CDCl_3$, 20 °C) δ 7.34-7.27 (comp., 5H), 5.46 (t, *J*=7 Hz, 1H), 5.35 (t, *J*=6 Hz, 1H), 4.5 (s, 2H), 4.02 (s, 2H), 3.99 (d, *J*=6.5 Hz, 2H), 2.16-2.1 (comp., 4H), 1.8 (s, 3H), 1.76 (s, 3H); $^{13}$C NMR (125 MHz, $CDCl_3$, 20 °C) δ 139.8, 138.4, 131.9, 130.3, 128.3, 127.8, 127.5, 122.4, 72.2, 66.3, 43.5, 31.9, 26.5, 23.4, 21.6; HRMS (FAB) calcd. for $C_{17}H_{23}ClNaO^+$: 301.1335 [M+Na]$^+$, found 301.1333 [M+Na]$^+$.

***p*-Toluene-*3,7,11*-trimethylundeca-*2E,6E,10*-trienyl sulfone 21:**

*trans,trans*-Farnesol (9.7 g, 44 mmol) in a mixture of dry diethyl ether (96 mL) and HMPA (30 mL) was added triphenylmethane (0.97g, 40 mmol). The mixture was cooled to -53 ºC and then added *n*-butyl lithium (1.59 M solution in *n*-hexane, 28 mL, 45 mmol) and subsequently a solution of *p*-toluenesulfonyl chloride (10 g, 52 mmol) in dry diethyl ether (50 mL). After warming to -24 ºC, the reaction mixture was added lithium chloride (2.7 g, 65 mmol) and then stirred at room temperature for 2 h, at which point the starting material and the intermediate was consumed. The reaction was added water (0.30 L) and partitioned between a water layer and an organic layer (*n*-hexane/*i*-propyl ether=1:1, 3 x 0.30 L). The organic layers were combined, washed with brine, dried over anhydrous magnesium sulfate, filtered, concentrated to give a residue containing *1*-chloro-*3,7,11*-trimethylundeca-*2E,6E,10*-triene as pale yellow liquid (14 g). $R_f$ 0.73 (*n*-hexane/ethyl acetate=5:1).

Sodium *p*-toluenesulfinate (12 g, 65 mmol) was added to a DMF solution (110 mL) of the residue (14 g) at 0 ºC. The reaction mixture was stirred and allowed to warm to room temperature for 19 h. The reaction was added sodium *p*-toluenesulfinate (1.9 g, 10 mmol) and then stirred at room temperature for another 2 d. At which point a chlorotriene was completely consumed, the mixture was added water (0.30 L) and then partitioned between a water layer and an organic layer (*i*-propyl ether, 2 x 0.30 L). The organic layers were combined, washed with brine, dried over anhydrous magnesium sulfate, filtered, evaporated to give a crude sample of **21** as a pale yellow liquid. Purification of this residue by silica gel column chromatography (*n*-hexane/ethyl acetate=7:1)

afforded *p*-tolyl-triprenyl sulfone **21** (16 g, 56% in 2 steps from farnesol). $R_f$ 0.68 (*n*-hexane/ethyl acetate=5:1); $^1$H NMR (500 MHz, $CDCl_3$, 20 °C) δ 7.75 (d, *J*=8 Hz, 2H), 7.33 (d, *J*=8 Hz, 2H), 5.2 (t, *J*=8 Hz, 1H), 5.1-5.04 (comp, 2H), 3.79 (d, *J*=7.5 Hz, 2H), 2.44 (s, 3H), 2.08-1.96 (comp., 8H), 1.68 (s, 3H), 1.6 (s, 3H), 1.59 (s, 3H), 1.33 (s, 3H); $^{13}$C NMR (125 MHz, $CDCl_3$, 20 °C) δ 146.2, 144.4, 135.8, 135.7, 131.4, 129.5, 128.5, 124.2, 123.3, 110.4, 56.1, 39.7, 26.7, 26.2, 26.1, 25.7, 21.6, 17.7, 16.2, 16; HRMS (FAB) calcd. for $C_{22}H_{31}O_2S^-$: 359.2050 $[M–H]^-$, found 359.2041 $[M–H]^-$.

***9*-(*R*/*S*)-*p*-Toluenesulfonyl-*3,7,11,15,19*-pentamethyleicosa-*2Z,6Z,10E,14E,18*-pentaen-*1*-ol benzyl ether 22:**

A solution of *p*-tolyl-triprenyl sulfone **21** (3.5 g, 9.6 mmol) in dry THF (20 mL)/HMPA (6.7 mL) was added *n*-butyl lithium (1.59 M solution in *n*-hexane, 6.7 mL, 11 mmol) over 5 min at -55 ºC. After stirring at -55 ºC for 15 min, a solution of *8*-chloro-diprenol benzyl ether **20** (2.7 g, 9.6 mmol) in dry THF (10 mL) was added to the reaction mixture, and rinsed with dry THF (2 x 5 mL). Then the reaction was stirred and allowed to warm to 10 ºC over 3.5 h. The reaction solution was diluted with brine (0.20 L) and extracted with *n*-hexane/*i*-propyl ether (1:1, 3 x 0.40 L). Organic layers were combined and then washed with brine, dried over anhydrous magnesium sulfate, filtered, and evaporated to give a crude **22** as a brown liquid (7.1 g). Purification of this residue by silica gel column chromatography (*n*-hexane/ethyl acetate= 5:1) afforded *9*-Ts-pentaprenol benzyl ether **22** as a pale yellow oil (5.0 g, 86%). $R_f$ 0.61 (*n*-hexane/ethyl acetate=5:1, double development); $^1$H NMR (500 MHz, $CDCl_3$, 20 °C) δ 7.72 (d, *J*=8 Hz, 2H), 7.34-7.27 (comp., 7H), 5.43 (t, *J*=7 Hz, 1H), 5.15 (s ,1H), 5.09 (t, *J*=7 Hz, 1H), 5.03 (s, 1H), 4.94 (d, *J*=10 Hz, 1H), 4.49 (s, 2H), 3.99 (d, *J*=7 Hz, 1H), 3.87 (dt, *J*=11, 3 Hz, 1H), 2.77 (dd, *J*=13, 2 Hz, 1H), 2.51 (t, *J*=12 Hz, 1H), 2.43 (s, 3H), 2.08-1.94 (comp., 12H), 1.73 (s, 3H), 1.68 (s, 3H), 1.6 (s, 3H), 1.59 (s, 3H), 1.58 (s, 3H), 1.19 (s, 3H); $^{13}$C NMR (125 MHz, $CDCl_3$, 20 °C) δ 145.1, 144.3, 139.9, 138.5, 135.6, 134.9, 131.4, 130.6, 129.3, 129.2, 128.3, 127.9, 127.8, 127.5, 124.2, 123.4, 122.2, 117, 72.1, 66.3, 63.5, 39.75, 39.68, 32.1, 29.7, 26.7, 26.4, 26.2, 25.7, 23.5, 23.4, 21.6, 17.7, 16.3, 15.9; HRMS (FAB) calcd. for $C_{39}H_{54}NaO_3S^+$: 625.3691 $[M+Na]^+$, found 625.3696 $[M+Na]^+$.

***3,7,11,15,19*-Pentamethyleicosa-*2Z,6Z,10E,14E,18*-pentaen-*1*-ol 23:** Lithium (0.70 g, 0.10 mol) in a flame-dried flask was cooled to -70 ºC and then added ethylamine (13 g, 0.29 mol). To this flask, a solution of *9*-Ts-pentaprenyl alcohol benzyl ether **22** (1.6 g, 2.6 mmol) in dry diethyl ether (4 mL) was added in a dropwise manner over 5 min, and then rinsed with dry diethyl ether (3 x 3

mL). The reaction mixture was stirred at -65 °C for 1 h, at which point the reaction was completed. Isoprene was added to the reaction solution until the color of the reaction solution changed from dark blue to yellow, followed by addition of methanol until the color disappeared. The mixture was diluted with water (0.10 L) and ectracted with *i*-propanol (100 mL). The organic layer was washed with brine, dried over anhydrous magnesium sulfate, filtered, and evaporated to give a crude residue (2.0 g). The residue was purified by silica gel column chromatography (*n*-hexanes/ethyl acetate=4:1) to yield pentaprenyl alcohol **23** as a pale brown liquid (0.80 g, 86%). $R_f$ 0.32 (*n*-hexanes/ethyl acetate=5:1); $^1$H NMR (500 MHz, $CDCl_3$, 20 °C) δ 5.46 (t, *J*=7.5 Hz, 1H), 5.13 (comp., 4H), 4.09 (s, 2H), 2.1-1.96 (comp., 16H), 1.74 (s, 3H), 1.7 (s, 3H), 1.68 (s, 3H), 1.61 (s, 3H), 1.6 (s, 6H), 1.19 (s, 1H); $^{13}$C NMR (125 MHz, $CDCl_3$, 20 °C) δ 139.8, 136.2, 135.3, 134.9, 131.3, 124.43, 124.43, 124.35, 124.2, 124, 59, 39.7, 39.7, 32.2, 31.9, 26.7, 26.6, 26.5, 26.3, 25.7, 23.44, 23.35, 17.7, 15.97, 15.96; HRMS (EI) calcd. for $C_{25}H_{42}O^+$: 358.3236 $[M+H]^+$, found 358.3237 $[M+H]^+$.

***p*-Tolyl-*3,7,11,15,19*-pentamethyleicosa-*2Z,6Z,10E,14E,18*-pentaenyl sulfone 24:** A solution of pentaprenyl alcohol **23** (0.61 g, 1.7 mmol) in dry diethyl ether (8.5 mL) was added triphenylmethane (40 mg, 0.17 mmol) and then cooled to -60 ºC. The reaction mixture was added HMPA (freshly distilled, 1.2 mL), *n*-butyl lithium (1.59 M solution in *n*-hexane, 1.8 mL, 2.8 mmol) and *p*-toluenesulfonyl chloride (0.59 g, 3.1 mmol). The reaction was allowed to warm to -30 ºC over 1 h and added lithium chloride (recrystallized, 0.14 g, 3.4 mmol) at -30 ºC, and then stirred and allowed to warm to room temperature overnight. The reaction mixture was poured into water (60 mL) and extracted with ethyl acetate layer (60 mL). The ethyl acetate solution was washed with brine, dried over anhydrous magnesium sulfate, filtered, and evaporated to give an orange liquid containing *1*-chloro-*3,7,11,15,19*-pentamethyleicosa-*2Z,6Z,10E,14E,18*-pentaene (0.99 g). This material was used for the next reaction without further purification.

The crude residue (0.99 g) was dissolved in dry DMF (4.5 mL) and added sodium *p*-toluenesulfinate (monohydrate, 0.61 g, 3.4 mmol) at room temperature. The reaction solution was stirred at room temperature overnight, at which point *1*-chloropentaene was consumed. The reaction solution was added water (60 mL) and ectracted with ethyl acetate (2 x 60 mL). The organic layers were combined, washed with brine, dried over anhydrous magnesium sulfate, filtered, evaporated to give a crude **24** as a brown liquid (1.2 g). Purification of this material by silica gel column chromatography (*n*-hexane/ethyl acetate=20/1) yielded *p*-tolyl-pentaprenyl sulfone **24** as a colorless oil (0.82 g, 98% in 2 steps from **23**). $R_f$ 0.39 (*n*-hexane/ethyl acetate=5:1);

$^1$H NMR (500 MHz, $CDCl_3$, 20 °C) δ 7.75 (d, *J*=8.5 Hz, 2H), 7.33 (d, *J*=8 Hz, 2H), 5.22 (t, *J*=7.5 Hz, 1H), 5.1-5.08 (comp., 3H), 4.97 (t, *J*=7.5 Hz, 1H), 3.78 (d, *J*=8 Hz, 2H), 2.44 (s, 3H), 2.08-1.97 (comp., 12H), 1.88 (m, 2H), 1.79 (m, 2H), 1.73 (s, 3H), 1.68 (s, 3H), 1.65 (s, 3H), 1.6 (s, 9H); $^{13}$C NMR (125 MHz, $CDCl_3$, 20 °C) δ 145.7, 144.4, 136.1, 135.9, 135.3, 134.9, 131.2, 129.5, 128.4, 124.3, 124.1, 123.92, 123.88, 111, 55.9, 39.69, 39.68, 32, 31.9, 26.7, 26.6, 26.5, 25.73, 25.67, 23.5, 23.3, 21.6, 17.7, 15.964, 15.955; HRMS (FAB) calcd. for $C_{32}H_{49}O_2S^+$: 497.3453 $[M+H]^+$, found 497.3453 $[M+H]^+$.

**_9_-(_R_/_S_)-_p_-Toluenesulfonyl-_3,7,11,15,19,23,27_-heptamethyloctaeicosa-_2Z,6Z,10Z,14Z,18E,22E,26_-heptaen-_1_-ol benzyl ether 25:**

*p*-Tolyl-pentaprenyl sulfone **25** (0.17 g, 0.35 mmol) in dry THF/HMPA (10:1, 2.5 mL) was cooled to -61 ºC and then added *n*-butyl lithium (1.59 M in *n*-hexane, 0.38 mmol) in a dropwise manner over 4 min. After stirring at -61 ºC for 15 min, a solution of *8*-chloro-diprenol benzyl ether **20** (0.12 g, 0.43 mmol) in dry THF (0.5 mL) was added to the reaction mixture in a dropwise manner over 3 min. The reaction was stirred at -61 ºC further for 90 min, at which point the reaction was completed. The reaction solution was then poured into brine (20 mL) and extracted with *n*-hexane/diethylether (1:1, 2 x 30 mL). The organic layers were combined, washed with brine, dried over anhydrous magnesium sulfate, and evaporated to yield a crude material (0.28 g). Purification by silica gel column chromatography (*n*-hexane/ethyl acetate= 20:1) gave the *9*-Ts-heptaprenyl alcohol benzyl ether **25** (0.23 g, 89%). $R_f$ 0.36 (*n*-hexane/ethyl acetate=5:1); $^1$H NMR (500 MHz, $CDCl_3$, 20 °C) δ 7.71 (d, *J*=8.5 Hz, 1H), 7.34-7.26 (comp., 7H), 5.43 (t, *J*=7.7 Hz, 1H), 5.2-5.1 (comp., 4H), 4.97 (d, *J*=10 Hz, 1H), 4.9 (t, *J*=5.5 Hz, 1H), 4.49 (s, 2H), 3.99 (d, *J*=7 Hz, 2H), 3.86 (dt, *J*=12, 12, 2.5 Hz, 1H), 2.72 (d, *J*=13 Hz, 1H), 2.48 (d, *J*=13 Hz, 1H) 2.42 (s, 3H), 2.08-1.96 (comp., 16H), 1.8-1.75 (comp., 4H), 1.73 (s, 3H), 1.68 (s, 3H), 1.65 (s, 3H), 1.64 (s, 3H), 1.6 (s, 12H); $^{13}$C NMR (125 MHz, $CDCl_3$, 20 °C) δ 144.9, 144.3, 140, 138.5, 135.8, 135.3, 135, 134.9, 131.3, 130.5, 129.3, 129.2, 128.3, 128.1, 127.8, 127.5, 124.3, 124.1, 124, 123.9, 122.1, 117.6, 72.1, 66.3, 63.3, 40, 39.73, 39.69, 32.2, 32.1, 31.96, 31.89, 30.1, 26.7, 26.6, 26.5, 25.68, 25.66, 23.6, 23.5, 23.4, 23.3, 21.6, 17.7, 16; HRMS (FAB) calcd. for $C_{49}H_{70}NaO_3S^+$: 761.4943 $[M+H]^+$, found 761.4941 $[M+H]^+$.

**_3,7,11,15,19,23,27_-Heptamethyloctaeicosa-_2Z,6Z,10Z,14Z,18E,22E,26_-heptaen-_1_-ol 26:**

Lithium (56 mg, 8 mmol) in a flame-dried flask was cooled to -56 ºC and then added ethylamine (1.74 g, 39 mmol). To this flask, a solution of *9*-Ts-heptaprenyl alcohol benzyl ether **25** (0.21 g, 0.28 mmol) in dry diethyl ether (1 mL) was added in a dropwise manner, and then rinsed with dry diethyl ether (3 x 0.3 mL). The reaction mixture was stirred at -56 °C for 1 h at which point the reaction was completed. Isoprene was added to the reaction solution at -56 ºC, followed by addition of methanol and brine (20 mL). The mixture was extracted with ethyl acetate (4 x 20 mL), washed with brine, dried over anhydrous magnesium sulfate, filtered and evaporated to give a crude residue (0.12 g). The residue was purified by silica gel column chromatography (*n*-hexanes/ethyl acetate=10:1) to yield heptaprenyl alcohol **26** as a colorless oil (0.10 g, 73%). $R_f$ 0.28 (*n*-hexanes/ethyl acetate=5:1); $^1$H NMR (500 MHz, $CDCl_3$, 20 °C) δ 5.45 (t, *J*=7.7 Hz, 1H), 5.12-5.09 (comp., 6H), 4.09 (d, *J*=6.5 Hz, 2H), 2.09-1.96 (comp., 24H), 1.74 (s, 3H), 1.69 (s, 9H), 1.68 (s, 3H), 1.61 (s, 3H), 1.6 (s, 9H); $^{13}$C NMR (125 MHz, $CDCl_3$, 20 °C) δ 139.6, 135.9, 135.31, 135.27, 135.1, 134.8, 131.1, 124.9, 124.8, 124.48, 124.46, 124.33, 124.2, 124.1, 58.9, 39.68, 39.66, 32.2, 32.1, 31.9, 26.7, 26.64, 26.56, 26.4, 26.3, 26.25, 25.7, 25.6, 23.39, 23.37, 23.3, 23.26, 17.6, 15.92, 15.91; HRMS (EI) calcd. for $C_{35}H_{58}O^+$: 494.4488 [M+H]$^+$, found 494.4485 [M+H]$^+$.

***3,7,11,15,19,23,27*-Heptamethyloctaeicosa-*2Z,6Z,10Z,14Z,18E,22E,26*-heptaen-*1*-ol bis(2-cyanoethyl) phosphate 27:**

Heptaprenyl alcohol **26** (34 mg, 69 μmol) and 1*H*-tetrazole (recrystallized from ethyl acetate, 18 mg, 0.27 mmol) were azeotropically dried with dry toluene (2 x 1mL), and dried further under a vacuum. The mixture was dissolved in dry THF (0.7 mL, freshly distilled from sodium benzophenone ketyl). Bis(2-cyanoethyl)-*N,N*-diisopropyl phosphoramidite (23 μL, 89 μmol) was added to the reaction at 0 ºC. The mixture was stirred at 0 ºC for 70 min and then added pyridine (20 μL, 0.24 mmol) and sodium periodate in two portions (19 + 10 mg, 89 + 44 μmol). The reaction was stirred at 0 ºC for 15 min, and then added ethyl acetate (4 mL). The mixture was then added sat. sodium sulfite$_{aq}$ (10 mL) in a dropwise manner. The mixture was partitioned between a water layer and an organic layer (10 mL). The resulting organic layer was washed with sat. sodium sulfite$_{aq}$ (2 x 10 mL), washed brine, dried over anhydrous magnesium sulfate, to give compound **27** as a colorless oil (45 mg). This compound was used for the next reaction without further purification. $R_f$ 0.36 (R-OP($OC_2H_4CN)_2$, *n*-hexanes/ethyl acetate=3:1), 0.06 (R-OP(O)($OC_2H_4CN)_2$, *n*-hexanes/ethyl acetate=3:1).

***3,7,11,15,19,23,27*-Heptamethyloctaeicosa-*2Z,6Z,10Z,14Z,18E,22E,26*-heptaene-*1*-ol**

**phosphate diammonium salt 6:**

Heptaprenyl bis(2-cyanoethyl) phosphate **27** (45 mg, ≤ 69 $\mu$mol) was dissolved in dry methanol (13 mL) and added sodium methoxide (17 mg, 0.32 mmol). The reaction mixture was stirred at room temperature for 23 h, at which point the reaction was completed (checked by LC-MS, negative-mode). The reaction solution was evaporated to remove methanol and the resulting residue was subjected to reverse-phase column chromatography (Wakosil $40C_{18}$, 20, 40, 50, 70, 80, 90, 95, 100% methanol in sat. $NH_4HCO_{3aq}$) to yield compound **6** as a white solid (26 mg, 54% in 2 steps from **26**). $R_f$ 0.75 (ethyl acetate/AcOH/water=10:2:1); $^1$H NMR (500 MHz, $CD_3OD$, 20 °C) δ 5.43 (t, $J$=7.7 Hz, 1H), 5.15-5.09 (comp., 6H), 4.42 (t, $J$=7.7 Hz, 2H), 2.11 (comp., 24H), 1.73 (s, 3H), 1.68 (s, 9H), 1.67 (s, 3H), 1.62 (s, 3H), 1.6 (s, 6H); $^{13}$C NMR (125 MHz, $CD_3OD$, 20 °C) δ 140.2, 136.5, 136.3, 136.2, 136, 135.9, 132, 126.18, 126.14, 125.9, 125.46, 125.42, 123.72, 123.65, 62.9, 40.88, 40.85, 33.31, 33.25, 32.9, 30.8, 27.8, 27.7, 27.6, 27.6, 27.5, 26, 23.84, 23.79, 23.77, 23.73, 17.8, 16.2; HRMS (FAB) calcd. for $C_{35}H_{58}O_4P^-$: 573.4078 $[M-H]^-$, found 573.4096 $[M-H]^-$.

**Selected NMR spectra and HPLC traces of compounds:**

$^1$H NMR spectrum of depsi lipid I **3** (600 MHz, $CD_3OD$, 22 °C)

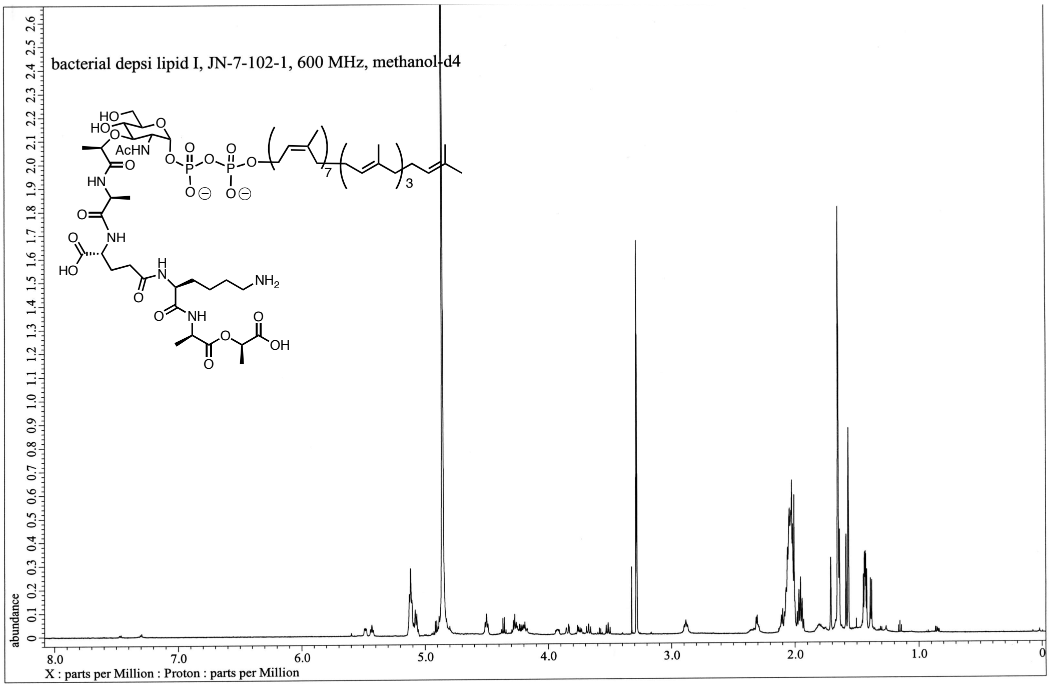

$^{13}C$ NMR spectrum of depsi lipid I **3** (150 MHz, $CD_3OD$, 22 ºC)

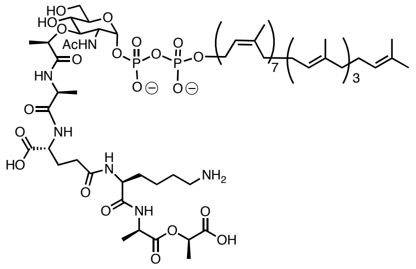

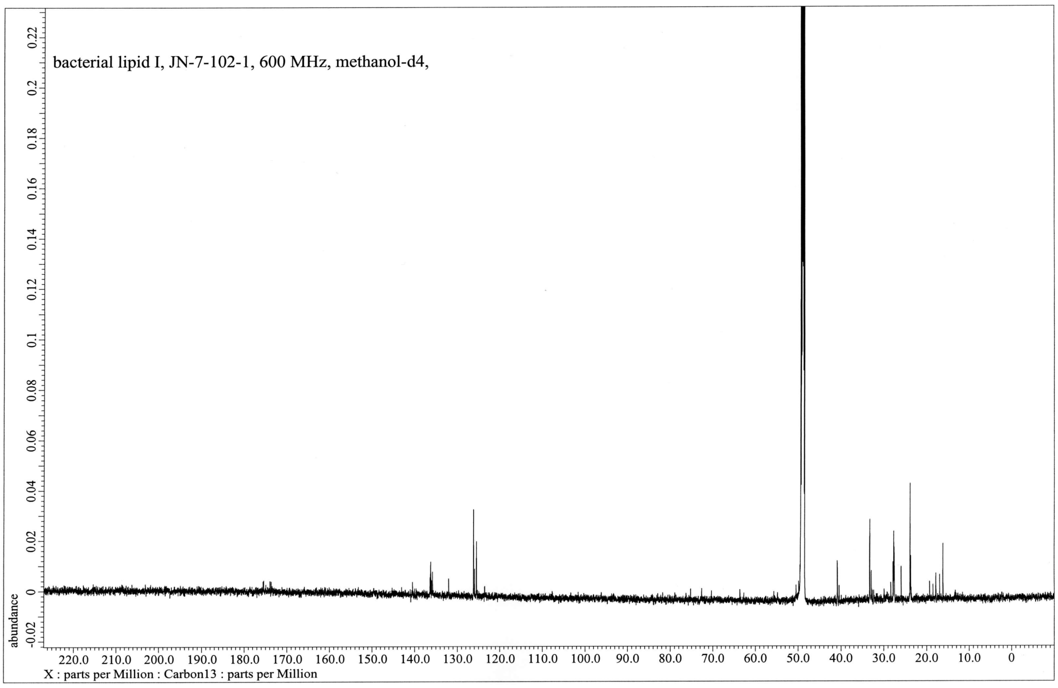

HPLC trace of depsi lipid I **3** (Analytical method II)

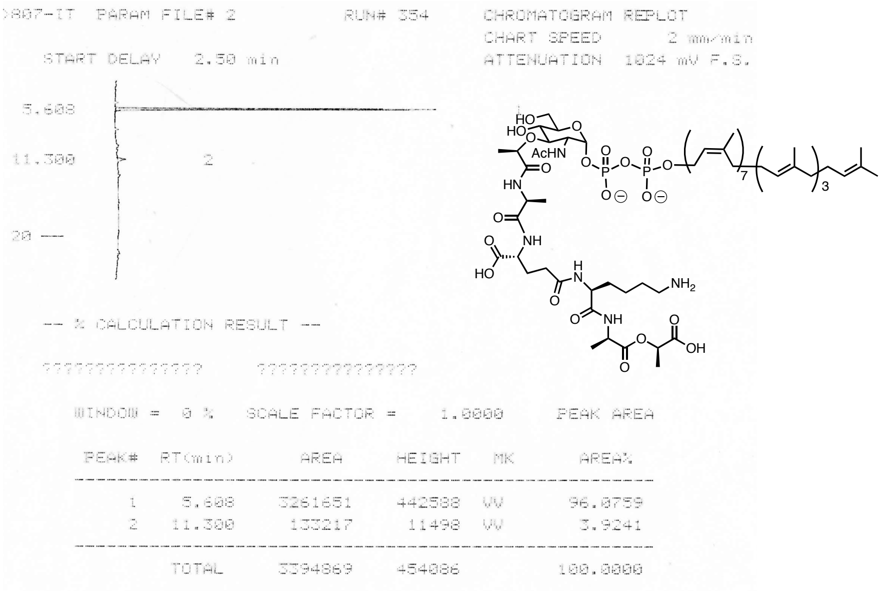


| PEAK# | RT(min) | AREA | HEIGHT | MK | AREA% |
|---|---|---|---|---|---|
| 1 | 5.608 | 3261651 | 442588 | VV | 96.0759 |
| 2 | 11.300 | 133217 | 11498 | VV | 3.9241 |
| | TOTAL | 3394869 | 454086 | | 100.0000 |

$^1$H NMR spectrum of depsi lipid I analogue **4** (500 MHz, $CD_3OD$, 20 ºC)

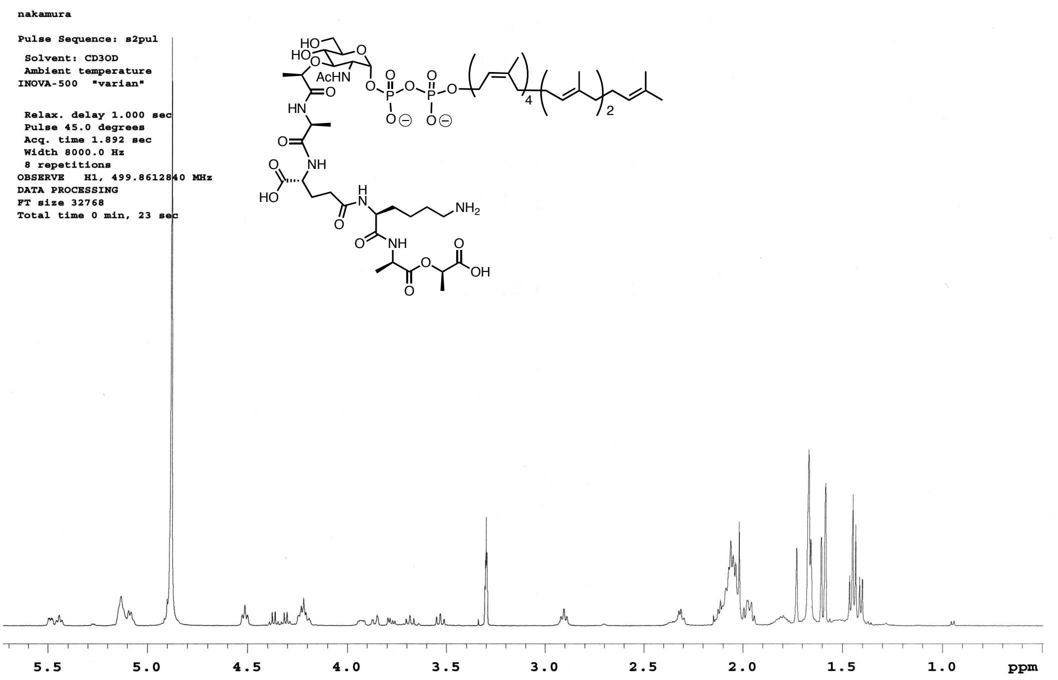

$^{13}C$ NMR spectrum of depsi lipid I analogue **4** (125 MHz, $CD_3OD$, 20 ºC)

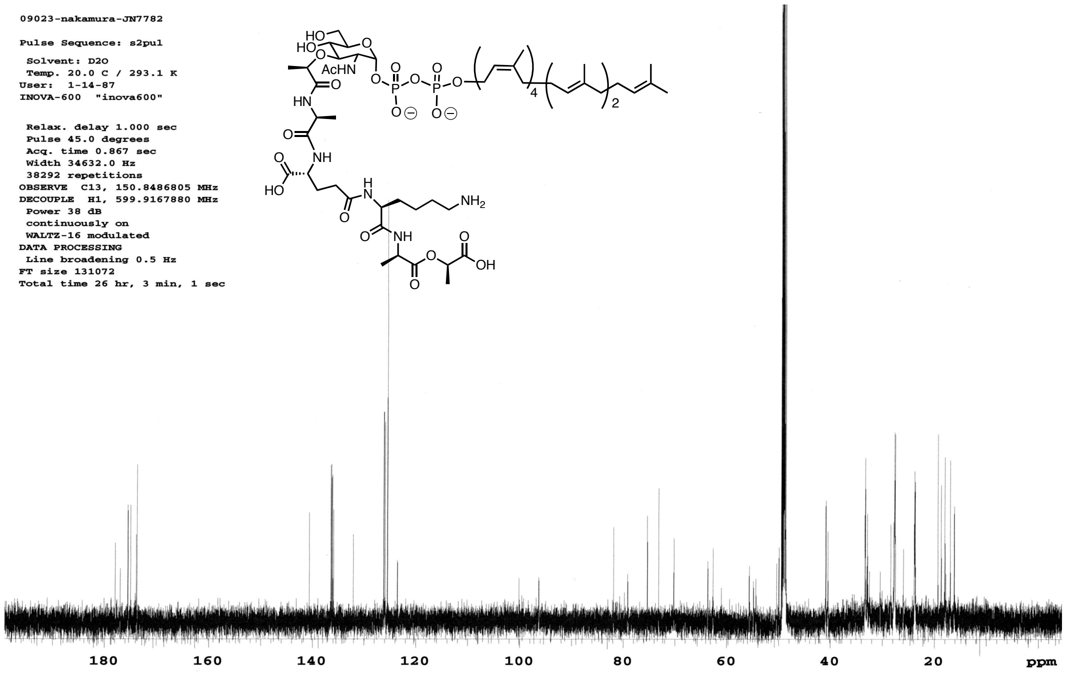


$^{31}P$ NMR spectrum of depsi lipid I analogue **4** (500 MHz, $CD_3OD$, 20 ºC)

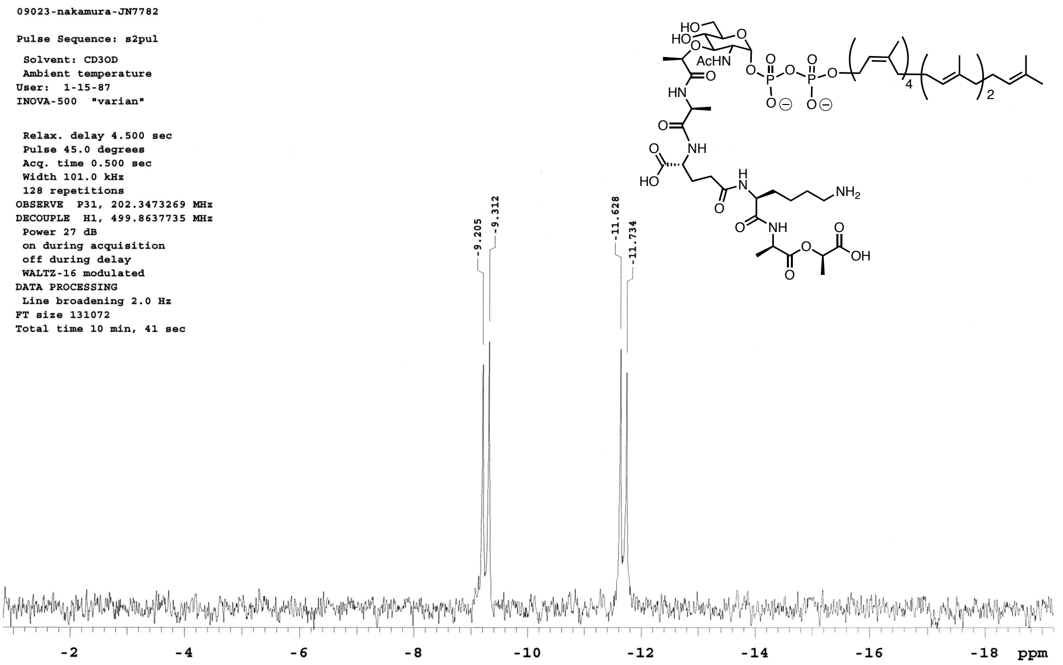

HPLC trace of depsi lipid I analogue **4** (Analytical method II)

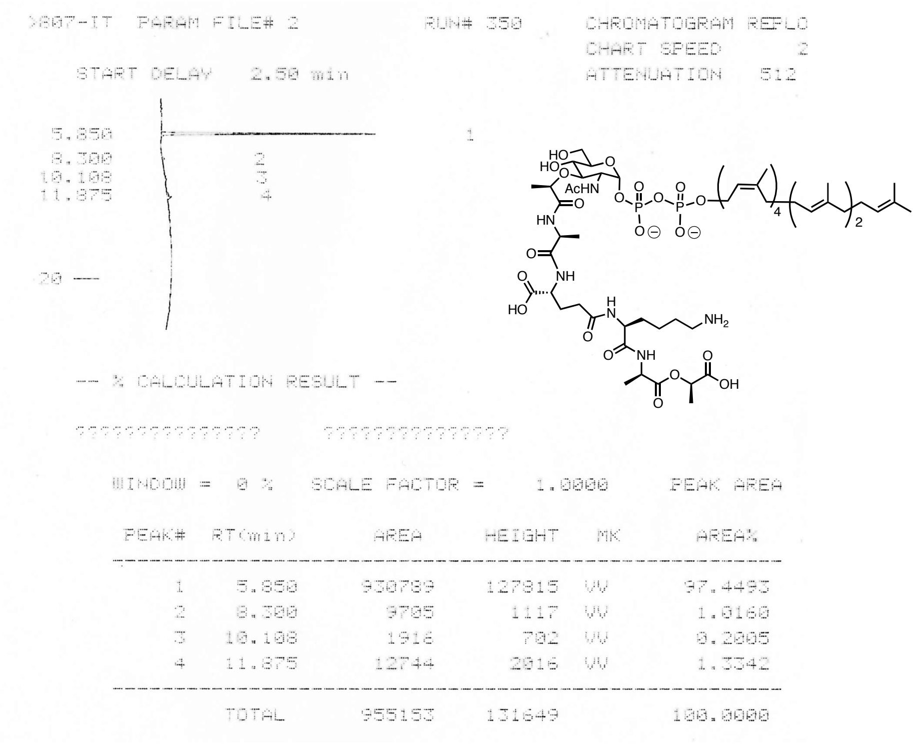

>807-IT PARAM FILE# 2 RUN# 350 CHROMATOGRAM REPLO

CHART SPEED 2

START DELAY 2.50 min ATTENUATION 512

5.850 1

8.300 2

10.108 3

11.875 4

20 —

-- % CALCULATION RESULT --

???????????????? ????????????????

WINDOW = 0 % SCALE FACTOR = 1.0000 PEAK AREA

| PEAK# | RT(min) | AREA | HEIGHT | MK | AREA% |
|---|---|---|---|---|---|
| 1 | 5.850 | 930789 | 127815 | VV | 97.4493 |
| 2 | 8.300 | 9705 | 1117 | VV | 1.0160 |
| 3 | 10.108 | 1916 | 702 | VV | 0.2005 |
| 4 | 11.875 | 12744 | 2016 | VV | 1.3342 |
| | TOTAL | 955153 | 131649 | | 100.0000 |

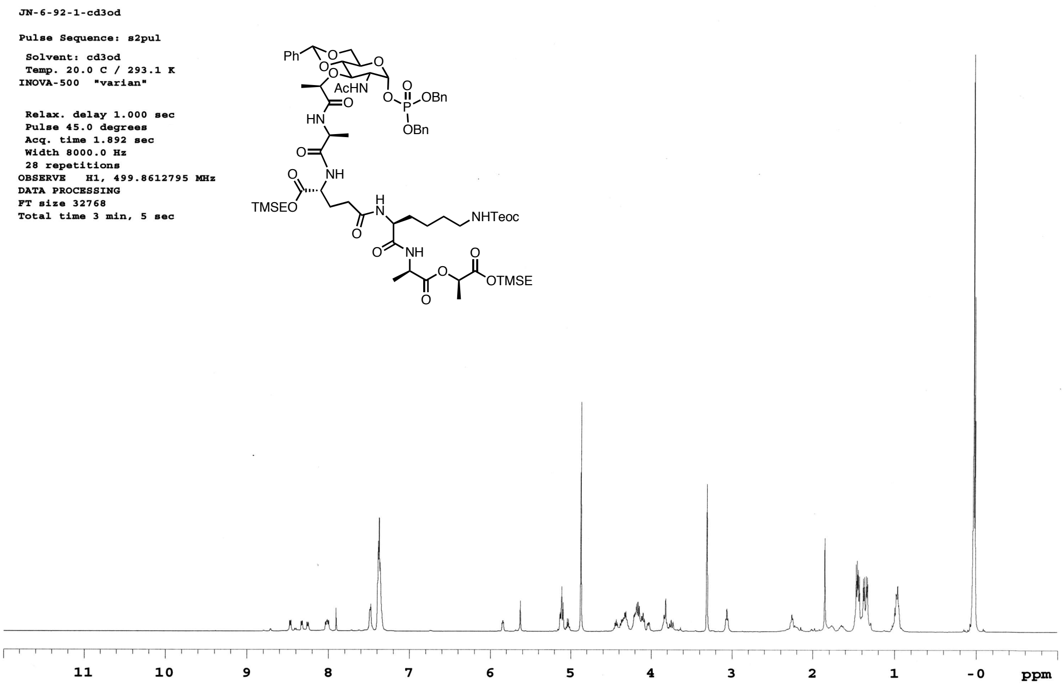


$^{1}$H NMR spectrum of Compound **7** (500 MHz, $CD_3OD$, 20 °C)

$^{13}C$ NMR spectrum of Compound **7** (500 MHz, $CD_3OD$, 20 ºC)

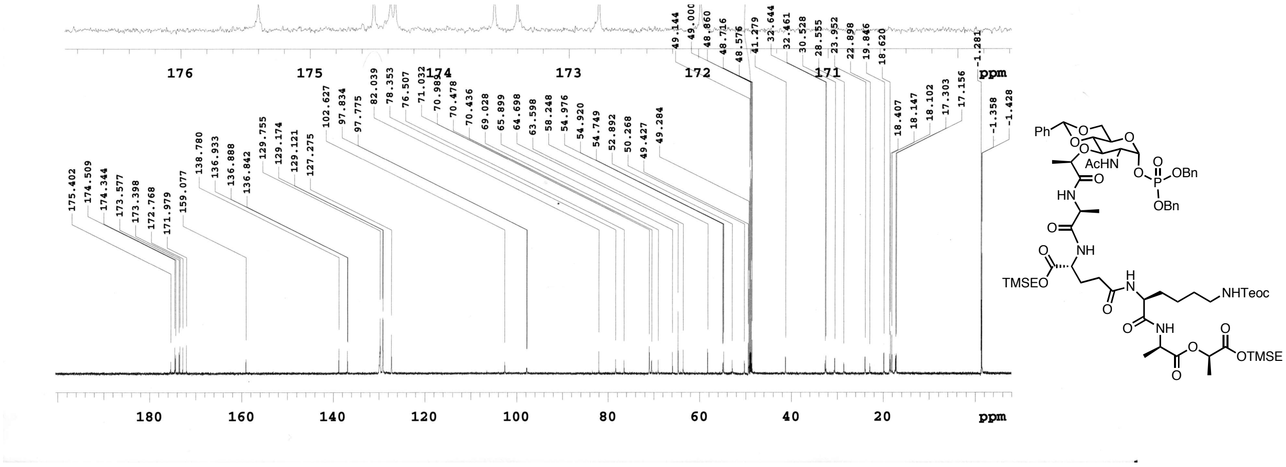


COSY spectrum of Compound **7** (600 MHz, $CD_3OD$, 20 ºC)

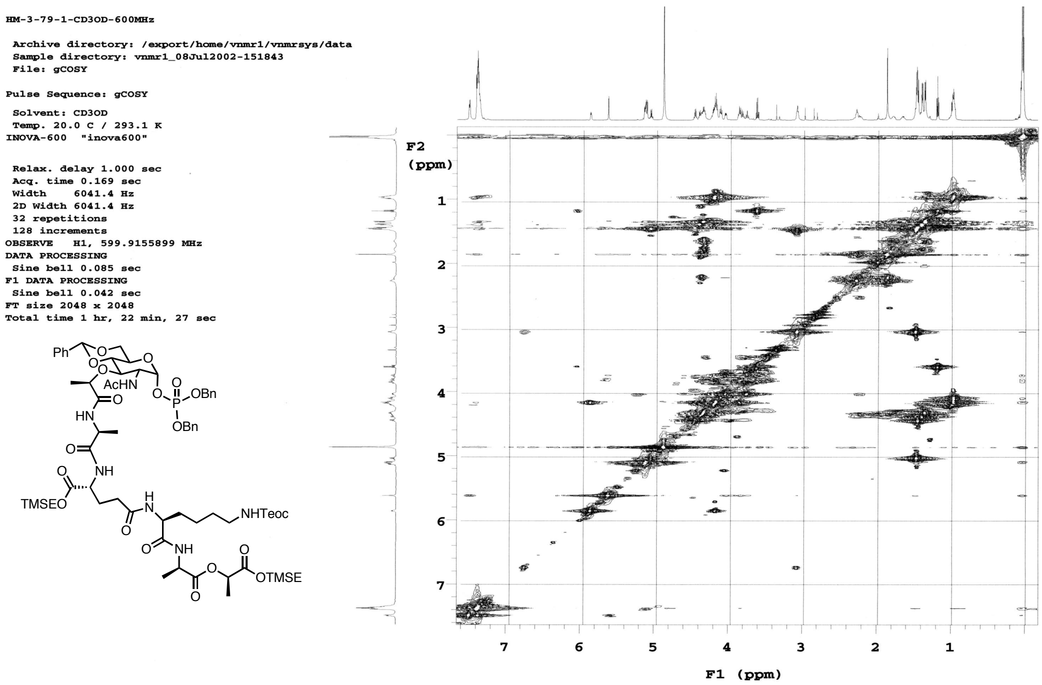

HMQC spectrum of Compound **7** (600 MHz, $CD_3OD$, 20 ºC)

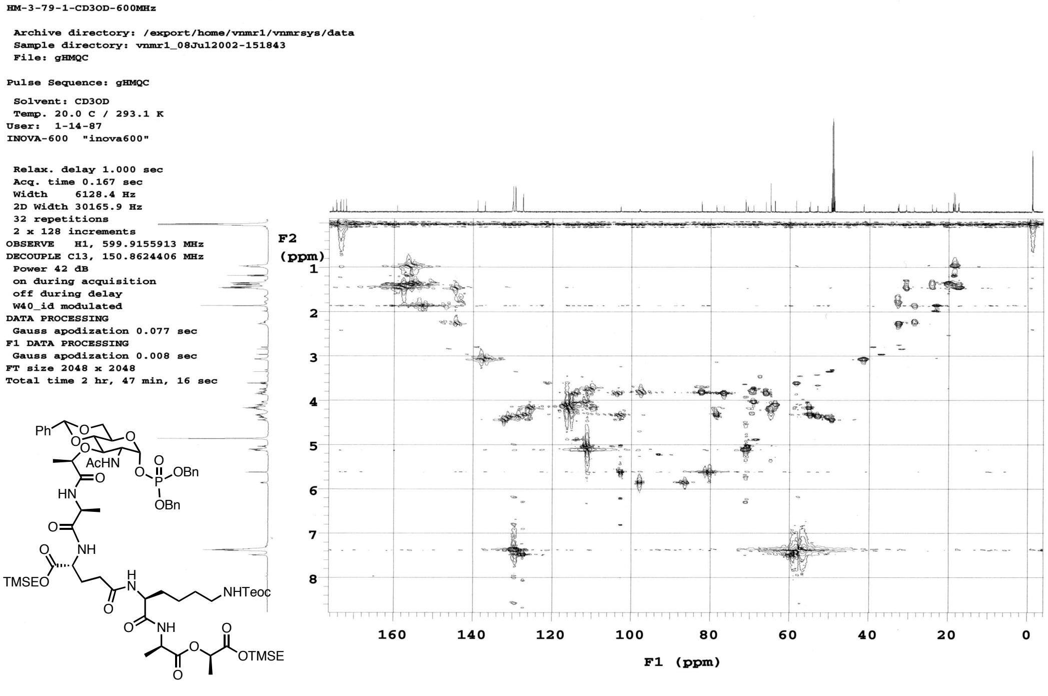


HMBC spectrum of Compound **7** (600 MHz, $CD_3OD$, 20 ºC)

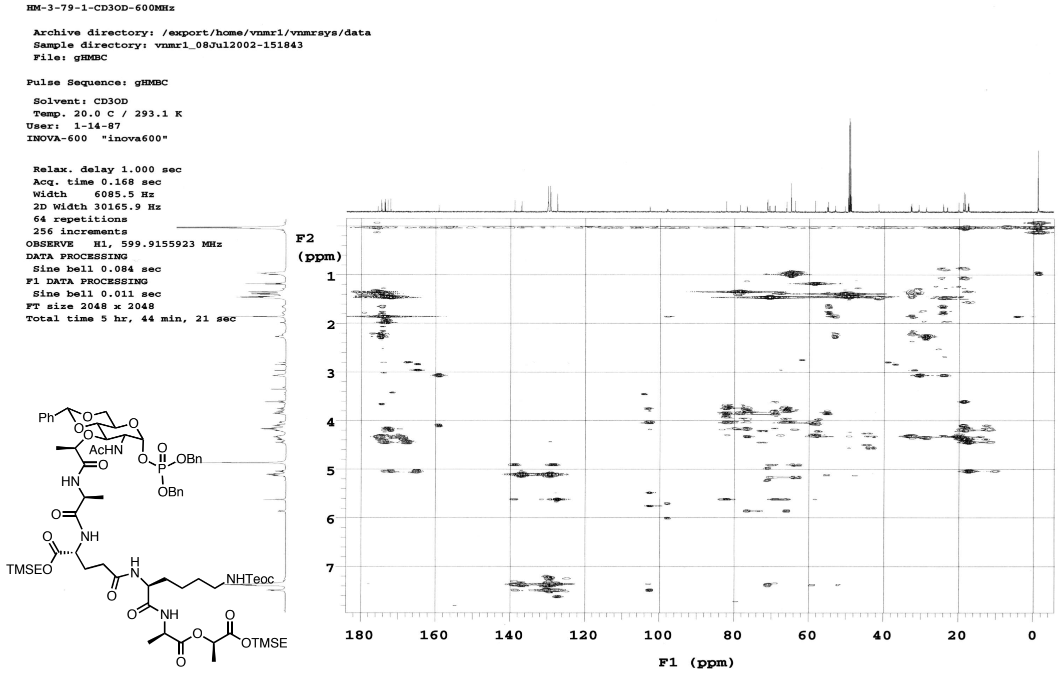

$^{1}$H NMR spectrum of Compound **6** (500 MHz, $CDCl_3$, 20 ºC)

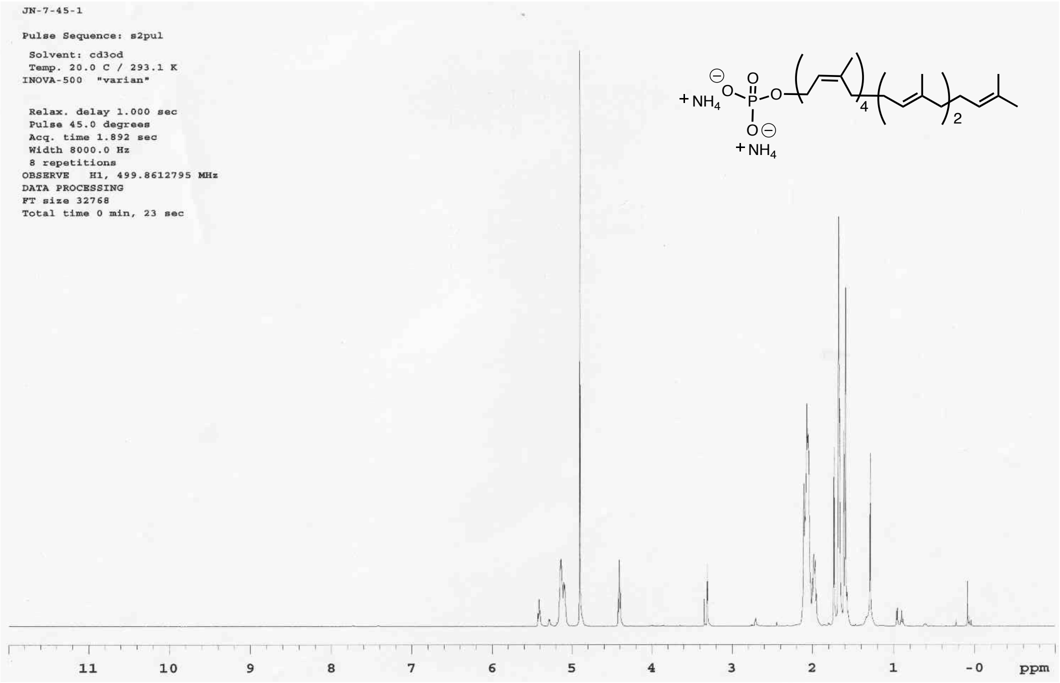


$^{13}$C NMR spectrum of Compound **6** (500 MHz, $CDCl_3$, 20 ºC)

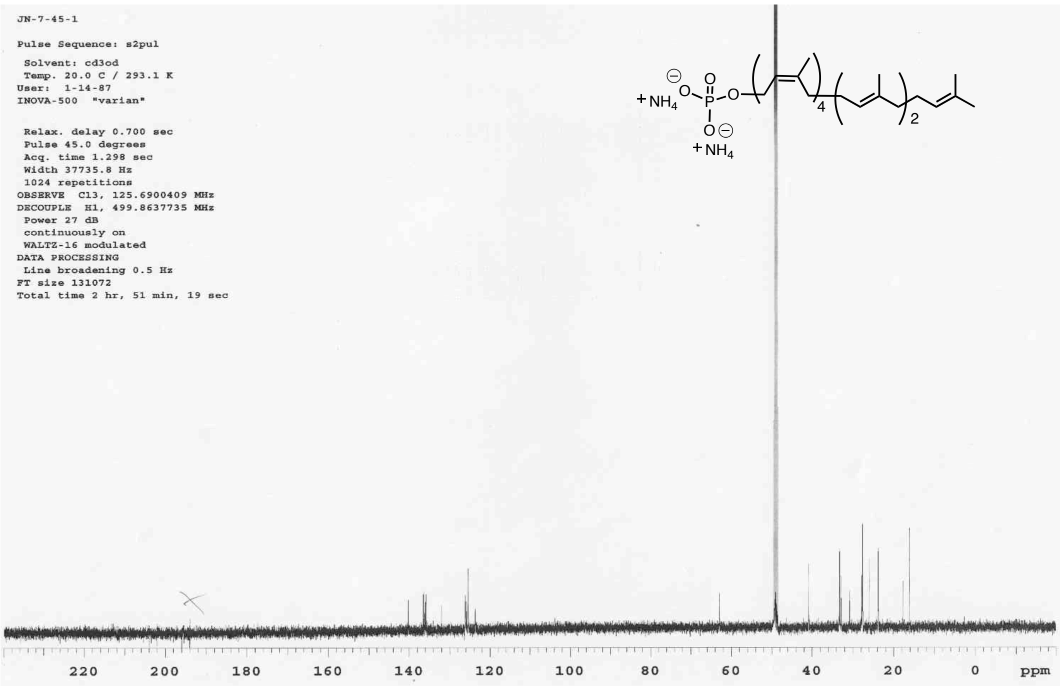

$^{1}H$ NMR spectrum of Compound **26** (500 MHz, $CDCl_3$, 20 ºC)

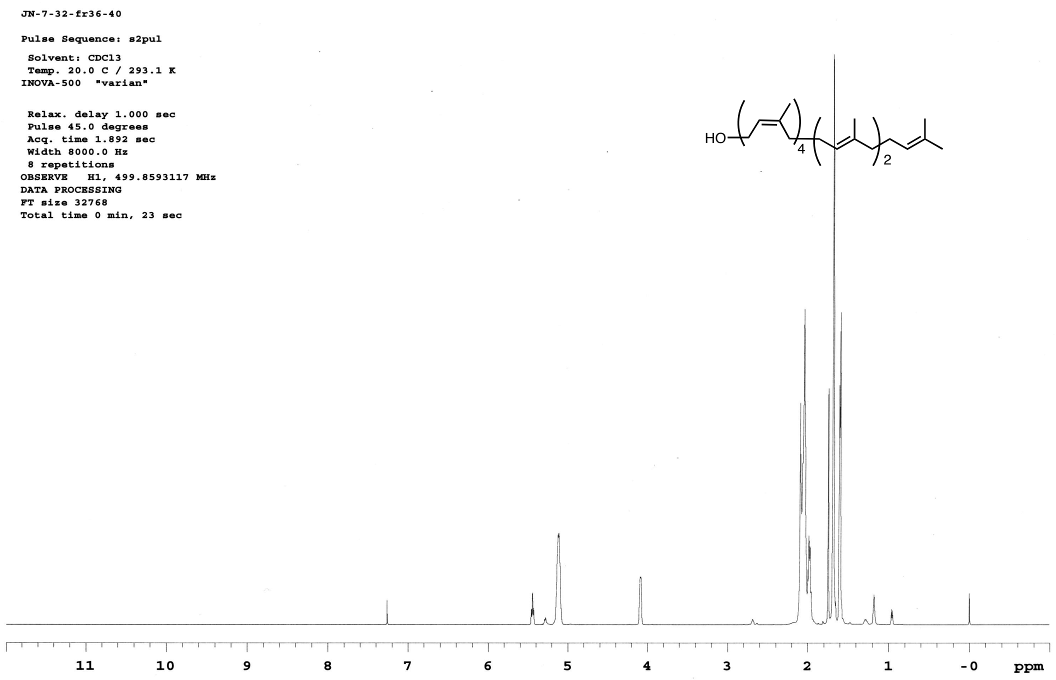


$^{13}C$ NMR spectrum of Compound **26** (500 MHz, $CDCl_3$, 20 ºC)

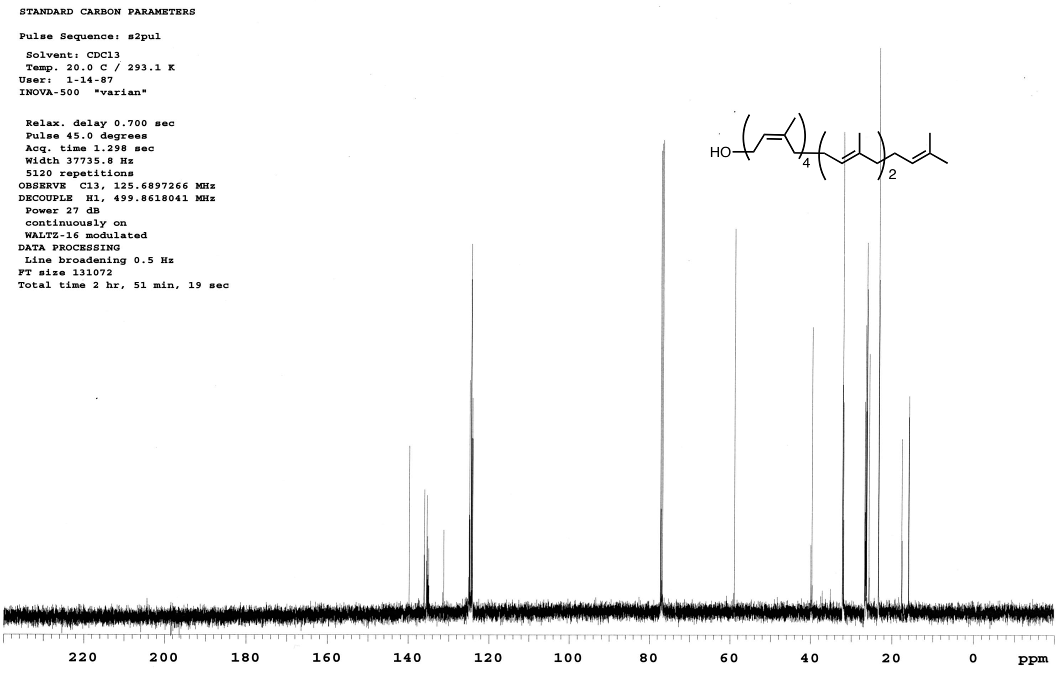

**Reference**

1 G. A. Roth, *J. Org. Chem.* **1995**, *60*, 8105.
